# Polyvagal theory: a journey from physiological observation to neural innervation and clinical insight

**DOI:** 10.3389/fnbeh.2025.1659083

**Published:** 2025-09-16

**Authors:** Stephen W. Porges

**Affiliations:** ^1^Traumatic Stress Research Consortium, Kinsey Institute, Indiana University, Bloomington, IN, United States; ^2^Department of Psychiatry, University of North Carolina at Chapel Hill, Chapel Hill, NC, United States; ^3^Department of Psychiatry, College of Medicine-Jacksonville, University of Florida, Jacksonville, FL, United States

**Keywords:** polyvagal theory, autonomic nervous system, vagus nerve, respiratory sinus arrhythmia, neuropeptides, neuroception, social engagement system, evolutionary neuroscience

## Abstract

Polyvagal theory (PVT) offers an integrative model of autonomic regulation that accounts for the evolution, neuroanatomy, and functional organization of the vagus nerve in relation to behavioral and emotional processes. This article revisits PVT by synthesizing its scientific foundations with recent advancements in transcriptomics, neurophysiology, and clinical application. Particular emphasis is placed on the theory's hierarchical model of the autonomic nervous system, the role of the ventral vagal complex in social behavior, and the construct of neuroception—the neural process by which safety and threat are detected without conscious awareness. The discussion incorporates both theoretical refinement and empirical validation while addressing common misconceptions and critiques of the model. In addition to the scientific narrative, the author offers a personal perspective on the intellectual and experiential origins of PVT, illustrating its translational value in clinical and therapeutic settings. By combining rigorous science with experiential insight, this article seeks to advance understanding of the autonomic foundations of social behavior and mental health.

## 1 Introduction

Polyvagal theory (PVT) emerged from my efforts to bridge psychological processes and autonomic function, drawing on insights from neurophysiology, neuroanatomy, clinical medicine, and the study of brain–body connections across disciplines. Developing this theory illuminated a fundamental challenge in science today: disciplinary silos often restrict collaboration and the integration of knowledge, as specialized methods and language can inhibit the exchange of ideas. When research remains isolated, advancing collective understanding becomes more difficult. This study examines the development of PVT and articulates its core principles in light of interdisciplinary engagement—particularly with colleagues unfamiliar with the theory's foundational literature. Bridging such gaps requires not only sharing knowledge but also cultivating openness to new perspectives, intellectual flexibility, and a spirit of curiosity about ideas that challenge established assumptions.

The development of PVT parallels the methodological approach advocated in “Strong Inference” (Platt, 1964), a paper I first encountered in graduate school. Platt advocated for a systematic approach to scientific investigation, emphasizing the design of experiments that test multiple, competing hypotheses. In many ways, the development of PVT embodies this methodology: Iterative hypothesis testing, informed by literature from diverse fields, has revealed the complex interplay between physiological regulation, health, and social behavior.

Although PVT may appear to be a tightly structured model, it was intentionally designed with flexibility. The theory was built to integrate new evidence rather than serve as a rigid, fixed framework.

At its core, PVT is anchored in foundational principles that have shown empirical consistency across diverse studies—particularly those addressing the phylogenetic and ontogenetic progression of autonomic nervous system (ANS) regulation and adaptive responses to states of illness, injury, and threat. By situating the theory within these evolutionarily conserved mechanisms, PVT offers a stable yet adaptable framework for scientific investigation. This structure invites rigorous empirical testing and theoretical expansion, enabling the development of biobehavioral hypotheses that connect autonomic regulation with health and behavior.

The development and dissemination of PVT have produced two notable outcomes. First, the theory has achieved broad uptake in mental health and clinical settings, where it has been cited extensively in peer-reviewed literature to inform research, assessment, and intervention. This reception suggests that PVT has offered a useful conceptual framework for integrating diverse biobehavioral phenomena.

Second, and perhaps more unexpectedly, a subset of critiques from biological and neuroscience disciplines has reflected persistent misinterpretations of PVT's core principles. Rather than engaging the theory's empirical foundations directly, some criticisms have centered on assumptions or claims not made by PVT. These misunderstandings have, in several cases, been reinforced through high-impact publications, underscoring the importance of maintaining rigor and accuracy in scientific communication. In the final section of this manuscript, I assess the status of these critiques and their implications, noting that many do not adhere to established standards of empirical challenge—such as direct engagement with the theory's hypotheses, logic, and published evidence.

This pattern underscores a broader responsibility within the scientific community: to promote accurate representation of foundational theories and ensure that emerging critiques contribute constructively to scholarly progress. Addressing misunderstandings has sometimes required redirecting focus from innovation to clarification. Nonetheless, this process has served to strengthen and refine the theoretical framework, reinforce methodological standards, and highlight the ongoing need for intellectual integrity in interdisciplinary discourse.

These experiences underscore a broader tension in the process of scientific advancement: the difficulty of developing innovative theoretical frameworks while responding to critiques that may not fully engage the requirements of hypothesis testing or theoretical coherence. Similar concerns were articulated by Platt (1964) and Popper (1959), whose emphasis on falsifiability and systematic disproof continues to inform modern standards of scientific evaluation.

Popper argued that scientific progress depends not on proof but on the generation of testable hypotheses that can be potentially refuted. In this view, robust theories are those that invite empirical challenge and remain open to revision. Platt extended this reasoning, advocating for “strong inference”—a methodological process that requires the articulation of alternative hypotheses and clear experimental tests.

For scientific discourse to fulfill these principles, critique must directly address a theory's stated claims, methodologies, and empirical basis. When discussions center on mischaracterizations or arguments not found in the original theory, the opportunity for empirical falsification is diminished. In such cases, researchers may find themselves spending disproportionate time clarifying existing positions rather than advancing new knowledge.

This concern is particularly salient in interdisciplinary fields, where conceptual translation can be complex and assumptions vary widely. It is essential that critiques distinguish between theoretical constructs and their interpretation, and that responses remain grounded in evidence, transparency, and mutual scholarly engagement. When this standard is upheld, critique becomes a vital driver of refinement and innovation, rather than an obstacle to progress.

This manuscript addresses these issues by situating PVT within its evolving scientific and conceptual context. It recognizes both the theory's contributions and the critiques it has elicited. Scientific advancement is sustained not merely through critique but through a commitment to intellectual rigor, methodological transparency, and engagement with the underlying logic of theoretical models. Accordingly, this study responds not only to specific empirical challenges but also considers a broader question: How can science maintain its progress when critiques shift away from evidence-based discourse and toward rhetorical simplifications?

Meaningful progress in the study of complex biobehavioral systems—such as the ANS—is not achieved through reductive argumentation, which oversimplifies dynamic, reciprocal, and context-dependent processes into isolated, linear causal relationships. Rather, it requires the careful evaluation of hypotheses, openness to alternative interpretations, and the ongoing refinement of conceptual frameworks that honor the hierarchical, interactive, and emergent nature of neurophysiological regulation. Reductive approaches risk obscuring the bidirectional feedback loops, developmental trajectories, and contextual contingencies that define systems such as the ANS, leading to misinterpretation and potentially counterproductive conclusions. In contrast, integrative models—such as PVT—prioritize coherence across levels of analysis and promote scientifically grounded understandings of how state regulation, behavior, and social engagement are dynamically interwoven. Thus, authentic scientific advancement rests on intellectual integrity, theoretical flexibility, and empirical fidelity, rather than on rhetorical reduction or conceptual simplification.

Since its original publication (Porges, 1995), PVT has evolved alongside scientific discovery, undergoing two major iterations (Porges, 2007a, 2023), culminating in *The Vagal Paradox: A Polyvagal Solution* (Porges, 2023) and *Polyvagal Perspectives: Interventions, Practices, and Strategies* (Porges, 2024). The latest iteration exemplifies the strategies of “strong inference,” systematically addressing contradictions in autonomic science and generating alternative hypotheses to elucidate the dual roles of vagal regulation. *The Vagal Paradox* integrates foundational evidence with empirical observation, advancing theoretical understanding while maintaining predictive and explanatory flexibility. Its methodology highlights the importance of robust theoretical frameworks and the necessity of iterative refinement through rigorous hypothesis testing.

When evaluated through the lens of strong inference, challenges to PVT have too often relied on strawman arguments and misrepresentation (e.g., Grossman and Taylor, 2007; Taylor et al., 2022; Grossman, 2023), rather than experimental evidence supported by plausible alternative hypotheses. This study aims not only to reaffirm PVT's principles but also to elucidate its foundational concepts, demonstrate its flexibility and clinical applications, and address persistent misconceptions. By fostering clarity and encouraging open, collaborative hypothesis exploration, the manuscript seeks to elevate the rigor and integrity of scientific discourse around PVT. Building on this commitment, the next section explores the early empirical observations and methodological challenges that gave rise to PVT.

## 2 Origins of polyvagal theory

PVT was introduced ~30 years ago to explain how autonomic state shapes reactivity in a complex and dynamically changing world (Porges, 1995). Although the theory has evolved over decades, its roots trace back to my graduate school years, when I unexpectedly observed beat-to-beat changes in heart rate variability (HRV) during an attention-demanding task. This observation sparked a cascade of questions about physiological mechanisms and function, ultimately guiding my lifelong pursuit of the interplay between physiology and behavior.

To fully appreciate this journey—and how contemporary scientific paradigms shaped PVT—it is important to understand the dominant models and assumptions that framed biobehavioral research in the mid-1960s and early 1970s. Two themes defined this era: (1) the constraints of hypothesis testing within rigid cause-and-effect experimental designs and (2) the pervasive assumption of psychophysiological parallelism—the idea that psychological processes have direct, one-to-one physiological signatures, regardless of anatomical or neuroanatomical level.

### 2.1 Methodological constraints and reframing the role of individual differences

In the 1960s and 1970s, psychophysiology was shaped by stimulus–response (S-R) paradigms, emphasizing direct mapping between stimuli and responses. Individual differences were minimized or treated as error, with researchers seeking to establish universal “laws of nature.” My own interests diverged from this focus on transient autonomic reactions to external stimuli. Instead, I was drawn to endogenous variability in beat-to-beat heart rate—a pattern now recognized as HRV.

By the early 1970s, I proposed that baseline HRV functioned as an intervening variable, offering predictive insights into an individual's health and biobehavioral repertoire. This perspective challenged dominant methodologies, advocating for a stimulus-organism-response (S-O-R) model in which the “organism” variable—autonomic state—could be meaningfully measured through HRV. This shift provided a new lens for understanding physiological regulation and behavior.

Remnants of the cause-and-effect model persist in medical research today, with randomized controlled trials still regarded as the gold standard. The pursuit of clear causal relationships has often led to studies with highly homogeneous samples, artificially restricting individual variability. Contemporary statistical approaches—including moderation and mediation models within regression frameworks—now permit the integration of individual differences directly into hypothesis testing. In contrast, during the 1970s, such variability was often dismissed as noise or a hallmark of “soft” science, limiting its perceived utility in formulating robust, testable hypotheses.

This bias was reinforced by the scientific culture of the time. As one National Academy of Sciences colleague bluntly informed me, science was about documenting “big effects”—so obvious that statistics would be unnecessary. Such attitudes reinforced the divide between fields that embraced tightly controlled experimental designs and those that prioritized the study of individual differences.

### 2.2 Psychophysiological parallelism and the emergence of polyvagal theory

A central challenge in psychophysiological research lies in the use of constructs that span disciplines. Historically, attempts to link psychological phenomena with physiological processes were shaped by psychophysiological parallelism—the belief that mental processes (e.g., feelings, emotions, and thoughts) have one-to-one neurophysiological signatures, independent of their neural level or anatomical origin. This view presumes that psychological states are expressed with uniform precision throughout the nervous system, often privileging cortical measures (e.g., EEG, evoked potentials, and fMRI) while neglecting the foundational roles of autonomic and brainstem function.

Anchored in the philosophical framework of psychophysiological parallelism, early psychophysiology often lacked an explicit neuroanatomical model, failing to acknowledge the hierarchical organization of the nervous system. Cortico-centric biases—dominant by the 1960s—have continued to shape research priorities, as noted by Cacioppo and Berntson (1994). These assumptions are reflected in contemporary initiatives such as the NIMH Research Domain Criteria (RDoC; Insel et al., 2010), which emphasize associations between psychological constructs and cortical circuits. While RDoC offers a dimensional and integrative model across levels of analysis, its practical implementation tends to privilege cortical correlates at the expense of subcortical and brainstem contributions to autonomic regulation and behavioral state. From the perspective of psychophysiological parallelism, this narrows the explanatory scope—overlooking how foundational neural circuits, particularly those involved in brainstem-visceral integration, co-regulate subjective experience and physiological reactivity. A more inclusive application of the RDoC framework would consider the parallel unfolding of embodied state and mental process, grounded in evolutionary neurobiology.

Polyvagal theory (Porges, 1995) emerged from this tradition but marks a decisive departure. Whereas the parallelism model—common in psychophysiological research—assumes that psychological constructs retain equivalent meaning across subjective, behavioral, and physiological domains (often seeking correlational “markers”), PVT emphasizes the hierarchical, integrative, and interactive organization of the nervous system. This reconceptualization echoes Hess (1949)'s foundational view in his 1949 Nobel Prize Lecture, *The Central Control of the Activity of Internal Organs*, where he opened with the assertion:

“A recognized fact which goes back to the earliest times is that every living organism is not the sum of a multitude of unitary processes, but is, by virtue of interrelationships and of higher and lower levels of control, an unbroken unity.” — Walter Hess, Nobel Lecture, December 12, 1949

By explicitly acknowledging the hierarchical organization of autonomic regulation, PVT provides a biologically grounded and evolutionarily informed framework for understanding the dynamic interplay between physiological state and psychological experience. This conceptual shift—from parallelism to hierarchical integration—sets the stage for a deeper examination of the evolutionary and structural innovations that underpin mammalian autonomic function.

### 2.3 Polyvagal theory: a hierarchical model of neural regulation

PVT outlines a hierarchical organization of neural regulation, deeply rooted in evolutionary and developmental principles. At the core of this framework is the concept that lower brain structures—particularly those controlling basic survival functions—must operate effectively before higher brain regions can support more complex behaviors such as problem-solving, social interaction, and creative thought. This evolutionary (phylogenetic) hierarchy is reflected both in developmental trajectories and in the functional progression of neural systems.

Higher brain structures—those responsible for language, cognition, and social engagement—emerged through structural and functional changes during vertebrate evolution. However, many cortico-centric and cognitive-centric models overlook the critical and ongoing role of lower brain mechanisms in regulating survival-oriented responses. These evolutionarily older neural systems, though repurposed in mammals for social communication and co-regulation, remain essential for managing stress responses whenever signals of threat are detected.

In contrast to the isomorphic assumptions of psychophysiological parallelism, PVT offers a hierarchical and integrative framework. It posits that neurophysiological processes supporting basic survival—regulated by foundational brainstem structures—must be reliably engaged before higher brain circuits can support complex behaviors and cognitive capacities. By integrating evolutionary, developmental, and functional perspectives, PVT provides a comprehensive account of the nervous system's organization. This model emphasizes the dynamic interplay between survival mechanisms and higher-order functions, underscoring the foundational role of brainstem and autonomic systems in regulating physiological states in response to environmental challenges—such as stress—that shape the conditions for social engagement, learning, and cognition.

## 3 Heart rate variability: a serendipitous observation and its scientific legacy

While conducting my Master's research, I made a serendipitous observation that would profoundly shape my scientific trajectory: Heart rate variability (HRV) markedly declined during a sustained attention task and then returned in a rhythmic, respiration-linked pattern after the task ended. At that time, the field lacked both an explanation for these fluctuations and a framework for interpreting their relevance to behavior, cognition, or neural regulation. This observation catalyzed my inquiry into the neurophysiological mechanisms underlying attentional engagement, vagal function, and the role of autonomic state in social behavior and adaptive co-regulation.

This early finding became central to my research agenda and ultimately informed the development of polyvagal theory. Later, the specific respiratory-linked rhythm in HRV would be identified as respiratory sinus arrhythmia (RSA), a non-invasive index of cardiac vagal tone. While RSA would become a valuable measure in studying state-dependent changes in neural regulation, it is not a core component of the theory itself. Rather, it served as an empirical bridge—offering insight into how fluctuations in vagal activity support cognitive flexibility, emotion regulation, and social engagement. These discoveries laid the foundation for exploring how the ANS dynamically adjusts to support adaptive behavior.

The publication of my Master's thesis (Porges and Raskin, 1969) marked the first peer-reviewed study to quantify HRV as a dependent variable linked to attention and mental effort. Building on this foundation, my dissertation research employed a reaction time paradigm to examine how individual differences in HRV relate to performance on cognitively demanding tasks. By randomizing the timing between the warning and response signals, I was able to separate the participant's reaction to the warning stimulus from the sustained, anticipatory attention required for a rapid response to unpredictable stimuli. My hypothesis was that unpredictability would maintain focused attention and suppress HRV—effectively compressing a physiological “spring” that stores potential energy for swift action. This suppressed HRV state, I reasoned, would support increased mental effort, leading to faster reaction times once the response was required.

During the design phase of my dissertation research, I proposed examining the relationship between individual differences in heart rate variability (HRV) and performance on attention-demanding tasks. At the time, the prevailing view in experimental psychology regarded the study of individual differences as lacking methodological rigor unless situated within designs that prioritized group-level comparisons. To address this concern and meet the expectations of my dissertation committee, a second reaction time paradigm was introduced in which the warning-response intervals were fixed and predictable. This experimental refinement preserved methodological control while enabling an exploration of how baseline HRV influenced both anticipatory and reactive performance. The approach contributed to a broader re-evaluation of HRV—from being treated as residual error to being recognized as a neurophysiological index of autonomic flexibility.

Despite considerable skepticism at the time, my dissertation research (Porges, 1972) was the first to demonstrate a direct relationship between HRV dynamics and cognitive performance. Specifically, I found that individuals who exhibited greater suppression of HRV during attention-demanding tasks—effectively compressing their physiological “coiled spring”—tended to perform better, achieving faster and more consistent reaction times. This finding provided some of the earliest evidence that the adaptive modulation of HRV reflects not only a physiological state but also the organism's readiness and capacity to meet cognitive challenges.

A subsequent publication (Porges, 1973) extended these findings, showing that higher baseline HRV was associated with more stable reaction time performance, particularly during tasks involving unpredictable timing demands. These studies established both baseline HRV and task-related changes in HRV as robust predictors of attentional performance and behavioral flexibility.

By demonstrating that individual differences in HRV are linked to the capacity for cognitive adaptation, this research set the stage for a new generation of studies connecting HRV to neurodevelopmental and clinical features—including ADHD, intellectual disabilities, mental health, and developmental trajectories (see below). This body of work ultimately helped shift the field's perspective, positioning HRV not as statistical “noise” but as a window into the dynamic regulation and resilience of the ANS.

### 3.1 Methodological innovation and scientific skepticism

The acceptance of these ideas, however, was far from immediate. At the time, studying individual differences in HRV—or in response patterns more broadly—was not widely considered a valid experimental approach within psychophysiology or experimental psychology. The prevailing attitude in both fields held that variability was a nuisance variable—a source of error to be statistically controlled, rather than a window into meaningful physiological or behavioral processes. Even today, many randomized clinical trials continue to regard individual differences as “noise,” overlooking their potential as indicators of adaptive capacity.

The prevailing reliance on group means, coupled with the routine dismissal of both individual and intra-individual response patterns, often led to the classification of outlying data as statistical noise or random error. While methodologically expedient, this practice risked obscuring meaningful variability—variability that may, in fact, reflect the adaptive flexibility of neural regulation. Within emerging frameworks such as PVT, these nuanced individual differences are increasingly understood as vital indicators of autonomic resilience. The methodological bias toward group-level conformity, shaped by a culture of procedural orthodoxy, frequently prioritized standardization over discovery. In retrospect, this orientation constrained the field's capacity to investigate the neural and behavioral signatures that underlie physiological regulation and adaptive functioning.

This skepticism toward individual variability was especially evident in the widespread dismissal of idiosyncratic data patterns. Despite these headwinds, my early research demonstrated that both baseline HRV and task-related changes in HRV were reliably associated with reaction time performance (Porges, 1972, 1973; Walter and Porges, 1976). These findings directly challenged prevailing assumptions, introducing HRV as a sensitive index of the nervous system's capacity for dynamic state regulation under cognitive demands. Importantly, HRV emerged as a plausible “intervening variable”—a physiological mediator linking psychological challenge to behavioral output—an insight that would later serve as a foundational tenet of PVT.

To test the generality of these findings and address ongoing skepticism, I sought new contexts in which to investigate the adaptive significance of HRV. After earning my PhD, I began my academic career as an assistant professor at West Virginia University, where I was privileged to conduct research in the university hospital's newborn nursery. At the time, few scientists had examined heart rate patterns in newborns, and the technical and conceptual challenges were considerable. Yet, I was deeply curious about whether HRV could serve as a marker of viability and resilience immediately following birth—a period marked by profound physiological transition and vulnerability. I wondered: could individual differences in HRV at this critical stage predict an infant's capacity to adapt, recover, and respond to environmental challenges? If so, this would provide strong evidence that HRV's role as an index of adaptive potential was not merely a product of experience or learned behavior but a fundamental feature of physiological regulation present from the very beginning of life.

Drawing from my experience measuring autonomic responses in adults, I adapted experimental methods for use with newborns by implementing rigorous methodological controls. Recognizing the critical role of biobehavioral state, I restricted testing to infants 24–72 h postpartum—allowing time for recovery from anesthesia and delivery-related stress—and conducted sessions exclusively during periods of quiet alertness, a state in which the nervous system is optimally poised to react to environmental stimuli. To ensure discrete measurement of each autonomic response, I designed stimulation paradigms with extended interstimulus intervals, minimizing the potential for overlapping or sustained reactions.

These design choices produced robust results. Newborns with higher baseline HRV showed greater heart rate responses to auditory and visual stimuli, anticipatory deceleration in conditioning paradigms, and more rapid recovery following stimulation (Porges et al., 1973, 1974; Stamps and Porges, 1975). These data provided early evidence that HRV was not merely an artifact of baseline variability but a measurable indicator of autonomic capacity and flexibility. The findings extended the relevance of HRV from adults and older children to the very beginning of life, highlighting its role as a fundamental biological feature of health and viability.

Despite the significance of these findings, they were initially presented as descriptive, lacking a clear neurophysiological explanation for how and why HRV was related to psychological and behavioral processes. At the time, the field continued to interpret such results through the lens of Wilder (1931)'s Law of Initial Values—the idea that the magnitude of a physiological response depends on the baseline value—leading many to dismiss these results as artifacts of baseline dependency rather than reflections of underlying regulatory mechanisms. The absence of a recognized neural model for HRV only reinforced these doubts. To address this entrenched perspective, I co-edited the volume *Psychophysiology* (Porges and Coles, 1976) which included a translation of Wilder's original paper. Our aim was to honor Wilder's historical contribution while inviting the field to critically re-examine its prevailing influence—especially considering emerging neurophysiological evidence that was reframing HRV, not as a mere statistical artifact but as a meaningful indicator of adaptive neural regulation.

Ultimately, these newborn studies helped reframe HRV as a biologically grounded index of autonomic resilience. Across both infant and adult populations, HRV consistently emerged as a predictor of attention, responsiveness, and recovery. These findings not only prefigured the later development of PVT but also validated the scientific importance of individual differences—transforming what was once dismissed as experimental “noise” into a signal of health, adaptability, and neural regulation.

## 4 Consequences and limitations of psychophysiological parallelism

The broader field of psychophysiology was historically limited by a lack of explicit neural models. In the absence of a robust framework specifying plausible neural pathways, researchers often relied on psychophysiological parallelism—attempting to correlate physiological variables directly with psychological constructs. This reductionist approach frequently underestimated the hierarchical and integrative role of central neural structures, particularly those within the brainstem, in regulating autonomic state. Consequently, autonomic reactivity was often misattributed to external stimuli or conscious processes, overlooking the foundational role of subcortical circuits in supporting homeostasis, adaptive behavior, and spontaneous shifts in physiological state.

A notable example of these conceptual shortcomings was Neal Miller's landmark study, published in *Science* (Miller, 1969), which claimed that autonomic responses could be modified through operant conditioning in anesthetized and paralyzed animals. His early findings generated considerable enthusiasm—some even speculated he might receive a Nobel Prize for demonstrating that the ANS could be trained using principles analogous to those used in behavioral conditioning. However, replication attempts failed, and with a more advanced neurophysiological understanding, it became clear that pharmacological agents used for paralysis, such as curare, likely disrupted vagal cholinergic pathways critical for heart rate regulation. In their attempt to eliminate motor confounds, Miller et al. inadvertently compromised the very neural circuits they intended to study.

During that period, prevailing psychophysiological and psychological paradigms prioritized responses to external stimuli, often marginalizing the study of internal regulatory processes. Physiological state regulation was typically considered secondary to externally driven responses. However, emerging evidence from HRV research began to challenge this assumption, demonstrating that fluctuations in cardiac rhythms could occur independently of discrete external cues. These findings pointed toward a more dynamic conception of the nervous system as an active modulator of internal state, rather than a passive receiver of environmental input.

Reflecting on this era, I encountered skepticism—even among prominent scientists—regarding the scientific merit of HRV. At the time, HRV was frequently dismissed as an artifact, grounded in the flawed premise that the heartbeat was inherently static unless altered by intentional behavior or external stimuli. What was often overlooked was that beat-to-beat variability in heart rate could emerge from endogenous neural mechanisms supporting homeostatic regulation. Without a neurophysiological model to contextualize HRV, such variability was often explained away as regression to the mean, a statistical anomaly per the Law of Initial Values, or attributed to insufficient experimental control. The dominant paradigm of psychophysiological parallelism—focused primarily on correlations between physiological and psychological variables—failed to account for the emergent, adaptive functions of neural regulation. It further reinforced the notion that learning principles governed autonomic activity, thus overemphasizing the role of conscious intent and external stimuli in shaping physiological state.

Retrospectively, these early conceptual limitations underscored the need for a theoretical model grounded in neurophysiology. The development of refined HRV metrics, along with a model of brainstem-mediated autonomic regulation, ultimately reframed HRV as a meaningful index of neural adaptability. This shift in perspective laid critical groundwork for the emergence of PVT, which redefined our understanding of the integrated regulation of behavioral, emotional, and physiological state.

## 5 HRV, mental effort, and the foundations of a neural framework

The early challenges in psychophysiology—rooted in assumptions of parallelism and cortico-centrism—highlighted a need for a framework that could explain how neural systems regulate behavior through quantifiable physiological processes. My early work with HRV provided the empirical foundations for such a shift.

In the late 1960s and early 1970s, HRV was often dismissed as unreliable or biological noise. Despite this skepticism, I remained committed to exploring its potential as a marker of physiological regulation and cognitive demand. In newborns, I observed that HRV reflected autonomic reactivity to environmental stimuli, which suggested that HRV might serve as a meaningful indicator of adaptive neural function. This insight became the first step toward conceptualizing HRV—and eventually RSA—as outputs of neurophysiological regulation.

At the time, individual differences in HRV were rarely considered meaningful. Many researchers, and even randomized clinical trials today, treat such variability as noise. However, in my dissertation work (Porges, 1972), I reported a correlation between baseline HRV and task performance: Individuals with higher HRV demonstrated greater autonomic flexibility and attentional stability. These findings led me to hypothesize that suppression of HRV could serve as an index of mental effort, with low variability reflecting heightened neural engagement and regulatory constraint.

This idea gained early support from Kahneman (1973), who cited my work in *Attention and Effort*. Observing reductions in pupillary oscillations during task engagement, he proposed that decreases in autonomic variability—such as diminished RSA or pupillary oscillations—reflected the mobilization of cognitive resources. Kahneman would later be awarded the 2002 Nobel Prize in Economic Sciences for his pioneering integration of psychological insights into economic theory, particularly regarding human judgment and decision-making under uncertainty. As he wrote:

“Porges (1972) reported that subjects who show the greatest reduction of cardiac variability during a task also tend to have the fastest reaction times… The reduction of autonomic variability during task performance is apparently a general effect…”

Kahneman's support helped position autonomic flexibility as a core mechanism underlying attention, intention, and motivation. Observed reductions in variability during cognitive effort reflect a neurophysiological adjustment—specifically, the temporary withdrawal of parasympathetic tone, primarily mediated by the ventral vagal complex, as discussed in detail below.

Importantly, this principle extends beyond mental effort. Illness, stress, and threat all trigger similar autonomic shifts—from a state of openness and restoration to one of defense and focused mobilization. These transitions deprioritize homeostatic functions such as growth and neuroplasticity in favor of survival. Viewed through a polyvagal lens, these shifts illustrate how brainstem circuits adjust physiological state to meet changing behavioral demands.

For decades, I was intrigued by the idea that spontaneous fluctuations in pupil diameter—particularly pupillary hippus—might serve as a non-contact analog to RSA, offering a dynamic, real-time window into autonomic regulation. Recent advances in technology have made this hypothesis empirically testable. Burtis et al. (2014) demonstrated that dynamic pupil behavior tracks fluctuations in brain state, while Schaefer et al. (2025) provided evidence that pupil diameter exhibits respiration-coupled rhythms—constricting during inhalation and dilating during exhalation—paralleling the respiratory-linked modulation of heart rate observed in RSA.

Although governed by distinct brainstem nuclei, the underlying mechanisms are strikingly parallel. RSA is mediated by myelinated vagal efferents originating in the nucleus ambiguus, whereas pupil constriction is controlled by parasympathetic fibers from the Edinger–Westphal nucleus that travel via the oculomotor nerve to the ciliary ganglion. Both systems are cholinergic, brainstem-mediated, and sensitive to oscillatory input from central respiratory-generating circuits, such as the pre-Bötzinger complex. This shared rhythmic entrainment suggests that respiration-linked fluctuations in parasympathetic tone can modulate both cardiac and ocular responses in a coordinated fashion.

## 6 Arousal theory: an antecedent to a neural framework

In the 1960s, psychophysiology emerged as a largely atheoretical and empirical field, dominated by the concept of arousal. Arousal theory conceptualized autonomic activity as a linear continuum from low to high activation, measured or inferred through behavioral and physiological responses. Performance was believed to follow an inverted U-shaped curve—optimal at moderate arousal and impaired at both extremes. The Yerkes–Dodson law (Yerkes and Dodson, 1908) formalized this relationship, casting arousal as the “energy” of the nervous system, observable through increased behavioral activity or physiological changes such as sweating and heart rate.

A later variant of this framework, the “window of tolerance” (Siegel, 1999), became widely adopted in mental health settings to describe the optimal range of physiological arousal for daily functioning. While metaphorical, this model has practical value for helping clients regulate emotions and maintain wellbeing.

Early psychophysiological research operated under the assumption that peripheral autonomic measures, such as electrodermal activity and heart rate, reliably reflected central arousal. These assumptions were shaped by limited knowledge of the ANS. Changes in these measures were typically attributed to sympathetic activation, reinforcing a model in which sympathetic activity was thought to mirror brain activation (e.g., Darrow, 1967). Researchers often inferred central nervous system processes from peripheral outputs, particularly from organs with sympathetic efferent innervation—sweat glands, blood vessels, and the heart—partly due to the ease of their measurement.

However, several critical factors were overlooked:

- The influence of parasympathetic (vagal) activity.
- The interactions between sympathetic and parasympathetic processes.
- The contribution of peripheral autonomic afferents.
- The role of central regulatory structures (e.g., brainstem).
- The adaptive and dynamic nature of autonomic function.
- The phylogenetic and developmental reorganization of brainstem circuits regulating autonomic function.

Prior to the 1990s, scientific knowledge was insufficient to move beyond arousal theory to a more integrated neurophysiological model. Arousal theory served as a placeholder—foundational for biobehavioral disciplines seeking to link brain and body indices to psychological processes. Over time, the field transitioned toward neuroendocrine models of stress, particularly those emphasizing the hypothalamic–pituitary–adrenal (HPA) axis. This shift redirected attention from fast-acting neural circuits to slower hormonal and molecular mechanisms.

By the late 1990s, cortisol had become a widely accepted operational definition of stress, a trend consistent with psychophysiological parallelism. While elevated cortisol levels were associated with poor health outcomes under chronic stress, the essential role of cortisol in mobilizing energy and sustaining endurance was often underappreciated.

In contrast, the development of a neurophysiological model centered on the autonomic nervous system required two key research advances:

1. Demonstrating that HRV functions as a global index of autonomic state, relevant to both mental and physical processes.
2. Developing new metrics sensitive to specific neural pathways embedded in HRV, particularly those reflecting vagal regulation.

Only with these advances could a comprehensive model of neural regulation—and, ultimately, PVT—emerge. This required a research agenda involving the stimulation and inhibition of neural pathways to the heart, refinement of HRV metrics, and investigation into the mechanisms underlying cardiac vagal control. The progression from observation to mechanism to theory depended on identifying physiological markers that could reliably reflect the neural circuits involved in emotion, health, and behavior.

PVT was developed, in part, to address the limitations of arousal theory by reframing the organization of autonomic state regulation. Rather than conceptualizing arousal as a unidimensional continuum, PVT introduces a hierarchical model rooted in the phylogenetic evolution of brainstem circuits. It identifies three distinct autonomic states—ventral vagal (associated with social engagement and calm), sympathetic (mobilization), and dorsal vagal (shutdown or immobilization)—each with its own neural architecture, adaptive functions, and behavioral correlates.

This reconceptualization represents both a theoretical advancement and a methodological shift. Central to its development was the refinement of HRV metrics to distinguish neural source specificity. For instance, updated approaches to quantifying RSA allowed for the isolation of vagal activity originating in the nucleus ambiguus—an essential step in anchoring autonomic state assessment within a neurophysiological framework.

Importantly, PVT does not dismiss the relevance of earlier models such as arousal theory or neuroendocrine frameworks centered on the hypothalamic–pituitary–adrenal (HPA) axis. Rather, it integrates these perspectives into a more mechanistically grounded and hierarchically organized model of autonomic regulation. Anchored in evolutionary neurobiology, PVT emphasizes the role of fast-acting brainstem circuits—particularly those of the ventral vagal complex—in dynamically regulating physiological state. These circuits are especially responsive in contexts of perceived safety and social interaction, enabling rapid, state-dependent shifts that support co-regulation, behavioral flexibility, and physiological resilience. In contrast, hormonal systems operate more slowly and systemically, serving complementary but distinct regulatory functions.

While some critiques contend that PVT disregards prior paradigms such as arousal theory or psychophysiological parallelism, the theory explicitly acknowledges and builds upon them. It incorporates their valuable insights while addressing their limitations—particularly the absence of neural specificity, the lack of evolutionary framing, and an oversimplified view of brain–body integration. In this way, PVT is not a repudiation of earlier models but a continuation of scientific progress toward a more biologically grounded and integrative understanding of autonomic function.

This trajectory culminated in the formulation of PVT as a unified framework—one that synthesizes decades of empirical research, methodological innovation, and conceptual refinement. Today, PVT serves as a foundational model for investigating resilience, adaptive behavior, and the neurobiological basis of mental and physical health.

## 7 From HRV to RSA—Establishing a neural perspective for polyvagal theory

The transition from traditional HRV research to the development of PVT was neither immediate nor linear. It required both empirical breakthroughs and conceptual innovation—a shift from interpreting HRV as statistical “noise” to understanding it as a neural signal originating from and dynamically regulated by brainstem circuits involved in vagal control. This scientific evolution unfolded through an ongoing dialogue with classical physiological literature, emerging data, and persistent methodological challenges.

A critical inflection point emerged with the identification of neural mechanisms mediating RSA—the rhythmic fluctuation in heart rate linked to the respiratory cycle—as a quantifiable index of myelinated vagal efferent activity originating in the nucleus ambiguus (NAmb). RSA would ultimately serve as a cornerstone for assessing autonomic state regulation and anchoring PVT in quantifiable neurophysiological metrics.

### 7.1 Historical foundations and the relevance of RSA

The conceptual origins of RSA trace back to 19th-century physiological inquiry. In 1847, (Ludwig 1847) documented fluctuations in heart rate associated with respiration, a phenomenon later examined in detail by Anrep et al. (1936a,b), who highlighted its reflexive and vagal components. Wundt (1902) noted a consistent temporal relationship between respiration and cardiac rhythm—acceleration during inhalation and deceleration during exhalation. Hering (1910) attributed this modulation to vagal influences, particularly in the context of reflexive respiratory control. Further conceptual contributions by Eppinger and Hess (1915) linked heightened vagal tone—vagotonia—to psychiatric conditions, presaging modern interest in RSA as a window into autonomic regulation and mental health.

These foundational insights established three enduring principles integral to the later formulation of PVT:

1. RSA is mediated by the vagus nerve, particularly its myelinated efferent pathways originating in the nucleus ambiguus.
2. RSA amplitude reflects the functional status of vagal efferent pathways to the heart, serving as a non-invasive index of parasympathetic cardiac regulation via the nucleus ambiguus (ventral vagal nucleus).
3. Elevated RSA is associated with adaptive emotional, behavioral, and health outcomes, including improved emotion regulation, social engagement, and physiological resilience.

Despite early recognition of its neural basis, 20th-century psychophysiological and clinical research often reduced HRV to statistical descriptors of system flexibility and health. While this abstraction proved useful in large-scale analyses, it frequently obscured the neurophysiological significance of RSA and its role in modeling the dynamic regulation of autonomic state. This trend persists today as HRV is routinely quantified for descriptive purposes—often without any intent to extract a neural (specifically vagal) component. The superficiality of this approach is further reinforced by the widespread use of consumer-grade wearables that provide metrics largely divorced from underlying autonomic mechanisms.

This reductive framing continues to shape contemporary psychophysiological and neurophysiological literature and dominates how HRV is reported by commercial devices, which rarely differentiate vagally mediated RSA from other sources of HRV. Considering this, RSA's relevance as a translational biomarker—anchored in neuroanatomy and evolutionary theory, as emphasized in PVT—warrants renewed emphasis and methodological clarity.

### 7.2 From abstraction to neurophysiology: reinstating the neural basis of RSA

PVT arose from a growing dissatisfaction with models of autonomic regulation that lacked anatomical specificity and evolutionary context. Prevailing paradigms—such as arousal theory or broad HRV metrics—were insufficient to parse the neural substrates responsible for behaviorally relevant physiological shifts.

The key insight was that the ANS is not a unitary axis, but a hierarchically organized system composed of three phylogenetically distinct platforms:

- **Ventral vagal complex (VVC)**—myelinated, supporting social engagement.
- **Sympathetic system**—supporting mobilization.
- **Dorsal vagal complex (DVC)**—unmyelinated, supporting immobilization.

This hierarchical architecture is not merely theoretical—it reflects an evolutionary sequence in which newer brainstem circuits inhibit older ones to support flexible, context-dependent behavior. Validating this model required a physiological metric capable of capturing vagal activity at its neural source, not just downstream heart rate fluctuations.

Building on the hierarchical model outlined in Box 1, it becomes essential to differentiate the distinct roles of the two vagal pathways—ventral and dorsal—in shaping physiological regulation and behavioral expression. While both pathways are parasympathetic, their functions diverge dramatically in terms of phylogenetic origin, neuroanatomy, and adaptive significance.

Box 1 **Adaptive autonomic dynamics and embedded hierarchical regulation.**

- **Autonomic hierarchy**: the ANS is organized as a phylogenetic sequence—VVC → Sympathetic → DVC.
- **Development mirrors phylogeny**: ontogeny recapitulates this sequence during postnatal maturation.
- **ANS as an intervening variable**: autonomic state mediates physiological regulation, behavioral expression, emotional reactivity, and health outcomes.
- **Jacksonian dissolution**: under threat, neural regulation regresses through the hierarchy—from VVC to DVC—reflecting conserved survival strategies.
- **Bidirectionality**: the hierarchy operates in both directions—regressing in response to illness or injury, and restoring through treatment, safety cues, and co-regulation.

The VVC, a mammalian innovation composed of myelinated fibers from the NAmb, coordinates the social engagement system and enables context-sensitive behavioral flexibility. It facilitates calm states by dynamically inhibiting older brainstem circuits, promoting co-regulation, and allowing the body to prioritize healing, growth, and restoration—all hallmarks of neurobiological safety. VVC activation supports immobilization without fear, evident in behaviors such as intimacy, nursing, and deep sleep, where homeostasis is preserved or enhanced.

In contrast, the DVC—evolutionarily older and composed of unmyelinated fibers from the DMNX—supports two functionally distinct immobilization strategies. When co-activated with the VVC, it contributes to homeostatic regulation through digestive efficiency and energy conservation. However, when ventral vagal tone is withdrawn—often in response to cues of danger or life threat—the DVC can dominate in a defensive mode. This leads to immobilization with fear, expressed as behavioral shutdown, fainting, or dissociation. Such withdrawal of VVC regulation reflects a biological shift from restoration to survival, consistent with PVT's operational definition of stress: the disruption of homeostatic processes due to loss of autonomic flexibility and instability in feedback circuits.

The contrasting roles of the ventral and dorsal vagal pathways become even clearer when their structural and functional features are viewed side by side. Table 1 organizes these distinctions into three adaptive modes—ventral vagal regulation, dorsal vagal homeostatic immobilization, and dorsal vagal defensive immobilization—linking each to its evolutionary origins, myelination status, target organs, functional roles, behavioral correlates, and clinical implications. By mapping these features in parallel, the table underscores how evolutionary history and neuroanatomy converge to shape specific physiological states and adaptive behaviors, and why differentiating immobilization without fear from immobilization with fear is central to both PVT and clinical application.

**Table 1:** Structural and functional differentiation of vagal efferent pathways according to polyvagal theory.

**Feature**	**Ventral vagal complex (VVC)**	**Dorsal vagal complex: homeostatic immobilization**	**Dorsal vagal complex: defensive immobilization**
Origin/nucleus	NAmb	DMNX	DMNX
Myelination	Myelinated fibers	Unmyelinated fibers	Unmyelinated fibers
Evolutionary age	Unique to mammals	Shared with ancestral vertebrates	Shared with ancestral vertebrates
Primary target organs	Above diaphragm (heart, bronchi, larynx, face)	Primarily below diaphragm (gut, kidneys, etc.); potential cardiac inotropic modulation	Systemic impact including heart and gut
Functional role	Supports social engagement, calm states, and homeostasis	Facilitates calm immobilization and restoration	Supports metabolically conservative defense during life threat (e.g., shutdown and collapse)
Behavioral correlates	Vocal prosody, facial expression, gaze, head orientation	Intimacy, nursing, digestion, restful bonding	Feigned death, dissociation, syncope, collapse
Physiological manifestations	Rapid heart rate inhibition via vagal brake, regulation of breathing and vocalization	Promotes digestive function, slow breathing, energy conservation	Bradycardia, apnea, digestive inhibition, hyporesponsivity
State supported	Calm social engagement	Immobilization without fear	Immobilization with fear
Response to threat	Withdraws rapidly under threat; re-engaged by safety cues	Supports calm state when VVC tone is present	Activates under overwhelming threat when VVC and sympathetic systems are suppressed
Regulatory context	Dynamic flexibility and co-regulation; supports social accessibility	Co-regulated immobilization contingent on safety cues	Unregulated autonomic collapse in absence of perceived safety
Cardiac influence	Fast, beat-to-beat inhibition of sinoatrial node (RSA modulation)	Slower modulation; contractility effects under specific conditions	Profound bradycardia, risk of syncope, especially in prematurity or trauma
Clinical implications	Enhances resilience, co-regulation, affective regulation, and therapeutic alliance	Supports digestion and recovery; associated with rest and safety	Associated with trauma, fainting, dissociation, autonomic shutdown

### 7.3 Quantifying vagal specificity

The challenge, then, was to re-anchor RSA within a rigorous neural framework. Traditional HRV metrics such as SDNN or high-frequency HRV lacked the specificity to isolate vagal contributions or track rapid autonomic shifts. RSA—when properly isolated—could offer a real-time index of cardioinhibitory vagal efferents originating in the nucleus ambiguus and terminating at the sinoatrial node.

Early RSA quantification methods, however, suffered from imprecision. Respiratory variability, baseline drift, and non-stationary noise all obscured the neural signal—especially in clinical populations such as preterm infants, where RSA amplitude may be diminished. These limitations made it difficult to validate RSA as a consistent neural index of parasympathetic regulation.

To address these challenges, we developed the Porges–Bohrer method, an analytic technique specifically designed to extract the vagally mediated component of RSA from complex physiological data reflected in HRV. Recognizing that beat-to-beat heart rate patterns are downstream expressions shaped by multiple neural and peripheral influences, the method removes low-frequency baseline trends and isolates respiratory-linked rhythms. This enables RSA to be interpreted not as a mere epiphenomenon of breathing but as a functional neural signature—reflecting dynamic regulation via myelinated vagal pathways.

Our hypothesis was clear: Vagal input to the heart should manifest as a rhythmic modulation in the heart period time series, with two defining characteristics:

- A frequency matching spontaneous respiration,
- An amplitude proportional to the strength of vagal efferent activity.

Meeting this analytic challenge was essential. The capacity to extract RSA from individuals with fragile, immature, or clinically compromised nervous systems—such as preterm infants, neonates, or patients with autonomic dysfunction—was a critical advance in developmental neuroscience. It enabled researchers to document vagal regulation during early life and under conditions of physiological vulnerability. This sensitivity to autonomic signals across developmental and clinical contexts was instrumental in establishing RSA as a robust neural biomarker and in laying the empirical groundwork for PVT.

### 7.4 From metric to model: RSA as a gateway to polyvagal theory

With this methodological reframing, RSA moved from the periphery of HRV research to the center of a new, neurophysiologically grounded model of autonomic regulation. The Porges–Bohrer technique transformed RSA from a vague marker of “variability” into a tool for investigating how brainstem circuits dynamically regulate physiological state in response to safety, danger, or life threat.

This allowed PVT to evolve beyond its origins in neonatal research and become a comprehensive model for understanding how the ANS supports behavior, emotion, and relationality. What began as an analytic innovation became the cornerstone of a theory describing how evolution shaped neural structures to enable co-regulation, trust, and resilience.

## 8 Methodological innovations and the foundations of polyvagal theory

### 8.1 Early insights and the limits of spectral analysis

In the early 1970s, while analyzing autocorrelations of sequential heartbeats, I noticed a repeating respiratory-linked rhythm embedded in the heart period signal. This observation pointed toward a physiological structure invisible to conventional statistical summaries. Around the same time, I served on a dissertation committee where a candidate applied spectral analysis to EEG data. It was during this defense that I realized frequency-domain decomposition could be applied to HRV to extract rhythmic neural inputs—specifically RSA.

This realization prompted a collaboration with Robert Bohrer, a mathematician on the committee and an expert in time-series analysis. Together, we adapted spectral methods to quantify RSA in heart period data. Initially promising, this effort also revealed significant challenges when applied to physiological signals.

### 8.2 The inherent challenges of spectral decomposition for RSA

Two major issues became apparent:

- Harmonic distortion: Physiological rhythms such as RSA are periodic but not purely sinusoidal. As a result, spectral decomposition produced a broad distribution of power across multiple harmonics rather than a sharp peak at the respiratory frequency. This distorted the identification of RSA and limited interpretability.
- Non-stationarity: Traditional spectral analysis assumes signal stationarity—an assumption rarely met in real-world physiology. Autonomic signals naturally vary with behavioral state, arousal, and context. When applied to non-stationary data, classical methods produced uns/or misleading results.

Rather than cleanly isolating RSA, spectral techniques often yielded blurred estimates due to smoothing and harmonic distortion that obscured dynamic vagal modulation. These limitations underscored that while spectral decomposition marked an important conceptual advance, it was insufficient for accurately characterizing RSA in the variable contexts typical of clinical, behavioral, or developmental research.

### 8.3 The Porges–Bohrer breakthrough: signal detrending for RSA extraction

A pivotal insight came in 1977 while browsing Kendall's *Time-Series Analysis* in a London bookstore. An illustration showing the use of local polynomial smoothing in economic data suggested an inverse application: removing slow-moving trends from physiological signals to reveal high-frequency components such as RSA. This led to the development of the Porges–Bohrer method.

We applied polynomial filtering to subtract baseline drift and isolate the respiration-linked vagal signal. This approach:

- Precisely defined RSA within the spontaneous breathing frequency band.
- Was robust to non-stationary baselines.
- Outperformed FFT-based and linear detrending techniques.
- Enhanced sensitivity to vagal modulation.

Empirical validation demonstrated its superiority over traditional peak-to-trough and HF-HRV methods (Lewis et al., 2012), which were vulnerable to distortion from respiratory rate variability and baseline shifts (e.g., Byrne and Porges, 1993).

### 8.4 Empirical validation and functional significance

#### 8.4.1 Animal studies

While a professor at the University of Illinois, my laboratory conducted research using the Porges–Bohrer method to validate RSA's neural origins:

- Vagal blockade suppressed RSA without affecting respiratory rhythm (McCabe et al., 1984).
- Baroreceptor activation increased RSA through reflexive vagal engagement (Yongue et al., 1982).
- Sympathetic blockade altered heart rate without affecting RSA, thereby confirming RSA's specificity as a parasympathetic index (Larson and Porges, 1982; Yongue et al., 1982).
- Developmental studies showed postnatal RSA increases reflecting vagal maturation (Larson and Porges, 1982).

#### 8.4.2 Clinical applications across the lifespan

- Neonates: RSA predicted survival and resilience more effectively than general HRV metrics (Porges, 1992).
- Neurosurgical patients: RSA predicted clinical outcome (Donchin et al., 1992).
- Children and Adults: RSA sensitively reflected real-time autonomic flexibility during challenge and recovery (Byrne and Porges, 1994; Porges et al., 1996).

Even in high-risk or unstable populations, such as during gavage feeding in high-risk newborns, the Porges–Bohrer method extracted meaningful RSA signals where other methods failed (Dipietro and Porges, 1991).

### 8.5 From descriptive variability to mechanistic insight

These advances repositioned RSA from a descriptive artifact of cardiorespiratory coupling to a dynamic neural signal—an operational index of brainstem-mediated vagal tone. This conceptual refinement established the empirical foundation for polyvagal theory (PVT). RSA emerged not merely as a marker of parasympathetic activity but as a functional signature of the ventral vagal pathway originating in the nucleus ambiguus—a real-time window into neural regulation of autonomic state. Building on this foundation, PVT posits that the vagal system evolved to support context-sensitive modulation of autonomic state, thereby facilitating social engagement, self-regulation, and physiological resilience.

## 9 Brainstem oscillators and the central generation of RSA

### 9.1 The common cardiopulmonary oscillator

Richter and Spyer (1990) identified a brainstem circuit—referred to as the *common cardiopulmonary oscillator*—that coordinates laryngeal, pulmonary, and cardiac functions. This oscillator integrates three core structures:

1. **Nucleus ambiguus (NAmb)**—source of myelinated vagal efferents regulating heart rate.
2. **Nucleus of the solitary tract (NTS)**—integrator of baroreceptor and pulmonary afferent input.
3. **Ventrolateral medulla**, including the pre-Bötzinger complex and phrenic premotor neurons—generator of respiratory rhythm and diaphragmatic activation.

These structures collectively coordinate a brainstem rhythm that synchronizes inspiratory drive, cardiac vagal outflow, and respiratory motor control—manifesting physiologically as RSA. Neurons in the pre-Bötzinger complex initiate respiratory rhythm and project to both the nucleus ambiguus and the phrenic motor nucleus, enabling precise temporal coordination of breathing, vagal gating, and cardiac deceleration (Smith et al., 1991; Feldman and Del Negro, 2006; Ashhad and Feldman, 2020).

This architecture supports a core tenet of PVT: RSA is not a passive mechanical byproduct of breathing but a measurable output of an evolutionarily conserved brainstem circuit that supports social engagement, homeostasis, and behavioral flexibility.

The coupling of the pre-Bötzinger complex, NAmb, and phrenic nucleus explains how changes in respiratory pacing—particularly the duration of expiration—can modulate RSA amplitude. However, such modulation should not be misconstrued as causal. Both RSA and respiratory rhythm arise from the same central oscillator; thus, RSA should be understood as a coherent neural output, not a mechanical artifact.

### 9.2 RSA as central output, not peripheral artifact

Building on Richter and Spyer's foundational work, subsequent studies (Dutschmann and Dick, 2012; Moore et al., 2013) confirmed that RSA originates from a central brainstem oscillator rather than from peripheral mechanical effects. While breathing—particularly slow-paced breathing—can modulate RSA amplitude, it does so by influencing the probability of vagal efferent activity in a phase-dependent manner not by generating RSA *per se*.

Inspiratory phases tend to suppress vagal output, while expiratory phases facilitate it. This phasic modulation enables respiration to function as a behavioral and physiological portal for flexible engagement of the vagal brake—a mechanism central to PVT. Crucially, this modulation represents a bidirectional feedback process within a loosely coupled neural circuit: Respiration shapes vagal influence, but RSA is generated centrally.

Physiological studies further clarify this mechanism. Vagal tone is typically inhibited during mid-to-late inspiration and increases during expiration (Iriuchijima and Kumada, 1964; Jewett, 1964; Katona et al., 1971). These effects are mediated by the respiratory–cardiac network involving the pre-Bötzinger complex, NAmb, and associated medullary feedback systems (Eckberg, 2003; Lopes and Palmer, 1976). Together, these findings affirm that RSA reflects a centrally generated, dynamically regulated vagal signal.

### 9.3 Reframing the debate: RSA as a neural signal

PVT interprets phase-dependent respiratory gating as evidence that RSA amplitude reflects the functional output of the ventral vagal complex. Rather than being dismissed as statistical noise or mechanical artifact, RSA is understood as a meaningful physiological signal of central vagal regulation.

This interpretation directly challenges the critique by Grossman and Taylor (2007), who argued that RSA is too confounded by respiration to serve as a reliable index of vagal tone. Their comparison of mammalian RSA to cardio-respiratory coupling in non-mammalian vertebrates—mediated by the dorsal motor nucleus of the vagus (DMNX)—fails to account for the evolutionary emergence of the mammalian ventral vagal complex. Unlike the DMNX, the NAmb is integrated within the common cardiopulmonary oscillator, coordinating phasic respiratory and cardiac activity within a mammalian-specific circuit.

In mammals, RSA is an evolutionarily derived output of the ventral vagal system, embedded within a neuroanatomical framework that supports sociality, co-regulation, and adaptive behavioral flexibility (Porges, 2021). This integration underscores RSA's functional role and evolutionary significance, as articulated in PVT. RSA thus serves as a non-invasive index of the dynamic regulation of the ventral vagal pathway—a biomarker of autonomic flexibility, emotional regulation, and physiological resilience.

### 9.4 Inspiration/expiration ratio as a modulator of RSA

Because vagal efferent activity is gated by respiratory phase, RSA amplitude increases when expiration is prolonged. This has been validated in both experimental and naturalistic settings:

- Strauss-Blasche et al. (2000) demonstrated that breathing patterns with shorter inspirations followed by longer expirations significantly enhanced RSA, independent of respiratory rate and tidal volume.
- Porges (2007a) found that individuals with a higher expiration-to-inspiration ratio exhibited greater RSA amplitude, even when controlling for other respiratory parameters.

These findings undermine the assumption that RSA must be “controlled” for respiratory confounds. Instead, they support a neurophysiological framework in which respiratory gating dynamically modulates vagal tone. RSA amplitude thus reflects a meaningful neural signature of autonomic flexibility, consistent with its interpretation as a central output of the ventral vagal system.

## 10 Developmental and lifespan trajectories of vagal regulation

### 10.1 Early life: vagal regulation in newborns and preterm infants

My research has long focused on the development of autonomic regulation in early life, especially in high-risk populations. Using the Porges-Bohrer RSA metric, we conducted studies showing that:

- Preterm infants consistently display lower RSA amplitude and reduced vagal efficiency compared to healthy full-term neonates (Porges, 1992; Porges et al., 1999, 2019).
- Maturation and clinical interventions—including enriched sensory stimulation and caregiver contact—enhance vagal function and increase RSA in these infants over time (Porges et al., 2019).
- These results establish RSA as a non-invasive biomarker of physiological resilience, helping to identify infants at risk and monitor their recovery trajectory. RSA amplitude reflects not just cardiac activity but the central regulation of biobehavioral state—a crucial indicator of the infant's ability to engage and adapt to environmental demands.

### 10.2 Later life: aging and autonomic flexibility

In collaboration with Jerome Fleg, a cardiologist at the National Institute on Aging, my research group conducted an experiment using participants from the Baltimore Longitudinal Study of Aging to investigate changes in HRV across the adult lifespan. The sample included normotensive adults aged 20 to 87, assessed during supine, seated, and standing postures (Byrne et al., 1996).

Our findings revealed that:

- Both RSA and low-frequency HRV (LF-HRV)—defined as spectral power in the 0.06 to 0.10 Hz range—declined significantly with age.
- These reductions were not significantly associated with aerobic capacity (peak VO_2_), body composition (BMI), or biological sex.
- Chronological age emerged as the primary predictor of HRV decline.

LF-HRV reflects a slower rhythm influenced by baroreflex activity—an autonomic feedback system regulating blood pressure through dynamic adjustments in heart rate and vasomotor tone. Afferent signals from arterial baroreceptors project to the nucleus tractus solitarius (NTS), which integrates this input and coordinates efferent output via sympathetic and parasympathetic pathways to maintain cardiovascular stability. As such, age-related attenuation of LF-HRV likely reflects diminished baroreflex sensitivity and reduced flexibility in central autonomic circuits governing blood pressure homeostasis. These findings suggest that age-related reductions in autonomic flexibility arise not solely from diminished cardiac ventral vagal tone (as indexed by RSA) but also from degraded reflexive control of cardiovascular function mediated by integrated brainstem-brain-body circuits.

## 11 Beyond RSA: revealing central mechanisms via weighted coherence

### 11.1 The weighted coherence: brainstem signaling of the common cardiopulmonary oscillator

The scientific foundations of PVT were significantly shaped by convergent findings, including the seminal work of Richter and Spyer (1990). Decades earlier, my laboratory's empirical exploration of respiratory–heart rate coupling in the mid-1970s anticipated core features of what would later be described by Richter and Spyer as the *common cardiopulmonary oscillator*—a brainstem circuit coordinating laryngeal, pulmonary, and cardiac functions.

Traditional statistical tools were inadequate for capturing the dynamic, rhythmic interplay among physiological systems. Time-series methods, particularly spectral and cross-spectral analyses, allowed for the identification of oscillatory components such as RSA and LF-HRV. Yet, variability in both RSA amplitude and respiratory patterns necessitated methodological refinement to resolve meaningful coupling.

To address this challenge, I collaborated with my colleague, mathematician Robert Bohrer, to develop a novel metric—weighted coherence—derived from cross-spectral analysis. This metric quantified the phase consistency between respiratory and heart rate signals, weighted by the proportional spectral power of heart rate at each frequency (Porges et al., 1980, 1981; Porges and Coles, 1982). Unlike RSA, which is modulated by peripheral vagal tone, weighted coherence indexed central integrative processes within the brainstem.

Importantly, while RSA and respiration may share a common frequency on average, biological rhythms are not perfect sine waves. They exhibit inherent variability, including phase jitter, reflecting the dynamic nature of neural regulation. Instantaneous fluctuations in respiratory phase relative to heart rate introduce variability in phase coupling, even when frequency alignment is maintained. Weighted coherence was specifically designed to account for this variability, capturing the consistency of phase alignment over time rather than assuming a rigid sinusoidal structure. This approach provided a more robust measure of central cardiopulmonary coordination, resilient to the natural variability of biological rhythms.

### 11.2 Functional implications and early evidence

Our early findings raised three foundational questions:

1. What individual features are associated with high or low coherence?
2. Does coherence mediate autonomic responsiveness to cognitive demands?
3. What neural mechanisms underlie these dynamics?

In children diagnosed with hyperactivity, we observed that low doses of methylphenidate (Ritalin)—those typically associated with enhanced cognitive performance—significantly increased weighted coherence, indicating improved integration of attentional and autonomic regulation (Porges et al., 1981). Notably, these coherence enhancements occurred without appreciable changes in RSA, suggesting that central brainstem mechanisms were engaged independently of peripheral vagal tone modulation. However, at higher doses, RSA was markedly depressed, and coherence declined, returning to pre-stimulus baseline levels. While these higher doses were effective in reducing disruptive behaviors, the physiological profile implies that behavioral control may have been achieved through suppression of vagal flexibility rather than its facilitation. This dose-dependent divergence highlights a potential trade-off: Higher pharmacologic doses may suppress outward symptoms while compromising neurophysiological adaptability, which is critical for sustained attention, emotional regulation, and social engagement.

In a separate reaction-time task (Porges and Coles, 1982), individuals with higher coherence demonstrated anticipatory heart rate deceleration prior to stimulus onset. This phenomenon was interpreted as a conditioned physiological response—a form of autonomic preparedness reflecting efficient central coordination.

These observations supported an emerging hypothesis: that weighted coherence reflects not peripheral vagal tone but rather the efficiency of brainstem mechanisms responsible for coordinating cardiopulmonary rhythms. While untested at the time via pharmacological blockade, the conceptual model pointed toward a central oscillator that would later align with Richter and Spyer's (1990) characterization of temporally integrated respiratory and cardiac nuclei.

### 11.3 Pharmacological dissociation: blocking the peripheral to reveal the central

A pivotal test emerged from a pharmacological study (Porges, 1986) utilizing atropine, a muscarinic cholinergic antagonist. As anticipated, atropine abolished HRV indices (RSA and LF-HRV) and elevated heart rate—consistent with peripheral cholinergic suppression. However, weighted coherence remained stable, even as vagal tone was pharmacologically silenced. Respiratory frequency was also unaffected.

These results collectively revealed a mechanistic dissociation:

- HRV metrics (RSA, LF-HRV) are mediated by peripheral cholinergic vagal pathways.
- Weighted coherence persists independently, reflecting central, non-cholinergic coordination mechanisms.

Though initially perplexing, these findings became coherent within the framework proposed by Richter and Spyer. Their single-unit cross-correlation recordings demonstrated temporally synchronized firing across key brainstem nuclei—particularly the NAmb and NTS—entrained to respiratory and cardiac rhythms. This neural architecture supported our interpretation: Weighted coherence provides a functional index of a central oscillator coordinating cardiorespiratory systems. Thus, rather than merely indexing peripheral vagal tone, coherence reveals the dynamic synchronization of brainstem centers—a phenomenon foundational to the autonomic organization articulated in PVT.

### 11.4 Weighted coherence in barosensory–heart rate coupling

To further probe brainstem regulation, we designed a baroreceptor-entrainment protocol using rhythmic tilt (Byrne and Porges, 1992). A motorized inversion table oscillated subjects at 0.08 Hz (12.5 s cycle), stimulating baroreceptors without overlapping with the primary frequencies of spontaneous breathing. Heart rate and tilt angle were synchronously recorded. Analysis showed a mean coherence of 0.54, with a phase lag of 4.7 s (SD = 2.4). These results paralleled the phase reported in earlier respiratory–heart rate studies and revealed a tilt dependent enhancement in LF-HRV independent of RSA, reinforcing that LF-HRV may be baroreceptor-mediated rather than sympathetically driven.

### 11.5 Orthostatic challenge and the “vagal paradox”

During sustained head-up tilt (70°), RSA decreased while LF-HRV remained stable (Hatch et al., 1986). The weighted coherence between blood pressure and heart rate increased in the LF range but declined at respiratory frequencies—suggesting a shift from respiratory-gated to baroreceptor-mediated cardiac control. This dissociation supports a revision of assumptions that interpret LF-HRV dominance in disease states as a marker of sympathetic activation. Our data instead point to a mixed autonomic state, marked by dorsal vagal involvement in baroreceptor regulation and concurrent ventral vagal withdrawal—a physiological configuration we later termed the “vagal paradox.”

This paradoxical state carries significant implications for cardiac function: persistent dorsal vagal tone, while preserving baroreflex integrity, may simultaneously impair myocardial contractility and electrical stability via mechano-electrical coupling. Such dynamics elevate the risk for arrhythmogenesis, particularly under orthostatic or stress-related challenges. Therefore, simultaneous monitoring of RSA, LF-HRV, and weighted coherence (i.e., between heart rate and blood pressure) provides a more comprehensive index of autonomic function—capturing both the shifting peripheral signatures and the central coordination of cardiopulmonary regulation. This integrated approach offers enhanced sensitivity to hierarchical vagal contributions and may clarify clinical phenotypes otherwise obscured by traditional autonomic indices.

### 11.6 Vagal efficiency: a dynamic marker of central regulation

The concept of the vagal brake describes how the ventral vagus modulates heart rate by inhibiting cardiac pacemaker activity. During challenge states, withdrawal of this vagal input allows for rapid cardiac acceleration. To quantify this mechanism, Bohrer and I developed a method for estimating RSA over short epochs (10–15 s), enabling the calculation of a dynamic slope—termed *vagal efficiency*—which reflects changes in heart period relative to RSA amplitude (Porges et al., 1999; Porges, 2025).

The concept of the vagal brake describes how the ventral vagal pathway dynamically regulates heart rate through rapid inhibition of cardiac pacemaker activity. During states of challenge or mobilization, withdrawal of this vagal input permits immediate cardiac acceleration. To quantify this mechanism, Bohrer and I developed what is now known as the Porges-Bohrer method, which allows for the estimation of RSA within short time epochs (typically 10–15 s). This methodological advance enabled the calculation of a dynamic slope—termed vagal efficiency—that captures the relationship between changes in heart period and RSA amplitude (Porges et al., 1999, 2019; Porges, 2025). By resolving RSA with fine temporal resolution, the Porges-Bohrer method provides an index of how efficiently vagal modulation adjusts cardiac output in response to changing physiological demands.

Vagal efficiency has also emerged as a predictor of treatment responsiveness: It forecasts outcomes to auricular vagal stimulation (Kovacic et al., 2020) and is itself enhanced by such stimulation (Kolacz et al., 2025). As a scalable, low-cost biomarker, it is well-suited for assessing central autonomic regulation and screening for dysautonomia. By quantifying the dynamic coupling between barosensory feedback and cardiac control, vagal efficiency offers a precise, functional index of the ventral vagal system's role in health and regulation.

Weighted coherence and vagal efficiency extend our ability to quantify central autonomic processes beyond RSA. Together, they provide functional insights into brainstem coordination of autonomic regulation, informing both basic science and clinical translational models of resilience and vulnerability.

## 12 Fetal heart rate patterns and autonomic resilience: a polyvagal framework

### 12.1 Autonomic foundations of perinatal stress responses

Before formally articulating PVT, I proposed that RSA in fetal and neonatal heart rate patterns could serve as a non-invasive marker of vulnerability during the stress of delivery and the transition to extrauterine life (Porges, 1979, 1988, 1992). Labor and delivery constitute a naturally occurring physiological stress test, challenging the ANS to maintain stability while adapting to a new environment. The dynamic fluctuations in fetal heart rate during this transition offer a real-time window into the nervous system's regulatory capacity.

By the late 1990s, I was able to evaluate this hypothesis directly within perinatology—the clinical field that originally index the theory. Applying the principles of PVT—especially the distinction between the two branches of the vagus nerve—enabled novel interpretations of fetal heart rate responses during labor (Reed et al., 1999).

### 12.2 Perinatal applications and core predictions of PVT

At the heart of PVT is the distinction between two vagal efferent systems. These systems—known as the ventral vagal complex (VVC) and the dorsal vagal complex (DVC)—differ in anatomy, phylogeny, function, and clinical relevance (see Table 1). The ventral vagus, originating in the NAmb, supports dynamic and adaptive autonomic regulation. It facilitates social engagement, state stabilization, and rapid recovery from stress via its influence on cardiac and respiratory rhythms.

- The dorsal vagus, arising from the DMNX, underpins metabolic homeostasis through unmyelinated fibers. During autonomic threat—such as intrauterine hypoxia—it may trigger energy-conserving immobilization, reducing metabolic demand through mechanisms such as bradycardia.

These distinctions inform several key predictions for fetal heart rate regulation during labor:

- Brief accelerations in heart rate reflect ventral vagal withdrawal to accommodate metabolic demand.
- When sympathetic activation is either inadequate to meet metabolic demands or becomes unsustainably prolonged, the ANS may shift to dorsal vagal dominance—manifesting as bradycardia—as a phylogenetically conserved strategy to conserve energy under extreme threat.
- In resilient fetuses, RSA rebounds post-bradycardia, signaling re-engagement of the ventral vagus.
- Persistent low RSA, especially following bradycardia, indicates chronic dysregulation and compromised adaptability.

### 12.3 From perinatal regulation to lifespan implications of trauma

The autonomic trajectories established during birth may offer a developmental blueprint for how the nervous system responds to future threat. Early disruptions—whether physiological (e.g., hypoxia) or relational (e.g., neglect)—can recalibrate vagal function and bias the system toward defensive states.

This recalibration may manifest as follows:

- Blunted RSA and reduced vagal efficiency, impairing adaptive social engagement.
- Over-reliance on dorsal vagal strategies, producing states of dissociation or withdrawal.
- Exaggerated or prolonged defensive responses, limiting opportunities for co-regulation and resilience.

These fetal patterns echo in trauma-exposed children and adults, where ventral vagal suppression becomes a barrier to psychological safety and relational healing (Kolacz et al., 2020; Dale et al., 2022). Understanding trauma through a Polyvagal lens—grounded in perinatal models—underscores the therapeutic importance of neuroception, co-regulation, and ventral vagal rehabilitation. Interventions that engage the social engagement system (e.g., face-to-face engagement, prosodic voice, and safe touch) serve as neural exercises to restore flexibility and resilience across the lifespan.

### 12.4 Empirical validation of polyvagal predictions during labor

Reed et al. (1999) tested these predictions in term fetuses using beat-to-beat heart rate data. They observed a consistent sequence:

1. Heart rate acceleration, reflecting ventral withdrawal and sympathetic activation.
2. Deceleration, indicating dorsal vagal engagement.
3. RSA rebound, marking ventral vagal reactivation in resilient fetuses.

This sequence illustrates the hierarchical recruitment of autonomic subsystems during labor. Critically, the reappearance of RSA after bradycardia served as a biomarker of adaptive capacity.

### 12.5 Bradycardia as a clinical marker of risk

While brief fetal decelerations are common, prolonged bradycardia is a clinical warning sign. It may signal hypoxia, acidosis, or poor perfusion—conditions requiring urgent intervention. From a Polyvagal perspective, bradycardia without RSA implies dominance of the dorsal vagal system, representing a shutdown physiology. Though adaptive in short bursts, this state is dangerous when prolonged. In the Reed et al. study, non-recovery of RSA predicted chronic fetal distress and autonomic inflexibility. In contrast, RSA rebound after bradycardia reflected ventral vagal integrity. These fetuses exhibited resilient cardiac–respiratory coordination, underscoring the role of vagal re-engagement in survival and recovery.

### 12.6 Implications for perinatal medicine and beyond

Labor offers a natural, high-resolution test of fetal autonomic resilience. Monitoring beat-to-beat heart rate patterns—particularly the interplay between RSA and bradycardia—provides a window into vagal maturation and adaptability.

These insights extend well beyond the delivery room:

- RSA and vagal efficiency may serve as early biomarkers of neurodevelopmental trajectory.
- Infants with low RSA recovery may benefit from targeted co-regulation interventions.
- RSA screening in NICUs or newborn exams could guide individualized care in at-risk populations.

Ultimately, the ability to flexibly regulate autonomic state in response to challenge is a core determinant of health. This principle—rooted in the autonomic orchestration of birth—scales upward across the lifespan to support trauma recovery, emotional regulation, and social connectedness.

## 13 From structure to function: evolutionary divergence in autonomic architecture

### 13.1 Evolutionary innovation in the vagus nerve

Polyvagal theory is grounded in an evolutionary principle: Mammals did not merely inherit the vagus nerve from earlier vertebrates—they transformed its structure and function to meet new behavioral demands, particularly those associated with social engagement. While reptiles and mammals share core vertebrate features, such as the vagus nerve and brainstem nuclei, the mammalian lineage underwent a profound autonomic reorganization.

This transformation is exemplified by the ventral migration and myelination of cardioinhibitory neurons from the DMNX to the NAmb. This evolutionary innovation gave rise to the VVC, a uniquely mammalian structure integrating autonomic regulation with cranial motor pathways supporting prosody, facial expressivity, and ingestion—forming the neuroanatomical foundation of the social engagement system (Porges, 2023).

### 13.2 Evolutionary origins of vagal specialization

From a now-extinct common amniote ancestor, two major branches emerged: synapsids (leading to mammals) and sauropsids (leading to reptiles and birds). This ancestor possessed a dorsal vagal system, originating in the DMNX, that mediated homeostasis through unmyelinated cardioinhibitory fibers.

Rather than representing a modification of modern reptiles, the emergence of mammals reflects a distinct evolutionary trajectory from this shared ancestor. Along the synapsid path, the NAmb evolved to accommodate myelinated cardioinhibitory output and integrate with cranial motor systems involved in communication and ingestion. This neurodevelopmental innovation supported the emergence of the social engagement system and marked a fundamental shift: from reflexive homeostatic control to a flexible system capable of regulating physiological state in service of social behavior—a feature absent in sauropsid relatives.

### 13.3 Structural continuity and functional innovation

Although the nucleus ambiguus (NAmb) is conserved across vertebrates, its functional role in mammals differs markedly. In reptiles and amphibians, it serves a primarily somatomotor role, innervating pharyngeal and laryngeal muscles for basic swallowing and vocalization. In mammals, however, the NAmb performs both somatomotor and visceromotor functions, contributing to cardiac regulation through myelinated vagal efferents that enable rapid, context-sensitive inhibition of the sinoatrial node.

This neuroanatomical innovation allows mammals to flexibly transition between states of mobilization and restoration in response to environmental and social cues, forming the physiological substrate for emotional regulation and prosocial behavior. The mammalian NAmb—core to the VVC—supports the social engagement system, enabling mammals to broadcast autonomic state through facial expression and vocal prosody. Such signaling fosters co-regulation, safety, and complex social communication.

The evolutionary divergence between reptiles and mammals represents more than an anatomical shift—it reflects a fundamental change in how autonomic state is regulated and integrated with social behavior. While reptiles rely primarily on unmyelinated DMNX efferents for cardioinhibitory control, mammals evolved myelinated NAmb pathways that support rapid, flexible heart regulation. This adaptation underpins a broader repertoire of behavioral strategies, integrating cardiac control with facial expression, vocal prosody, and other social signaling mechanisms.

Table 2 summarizes these distinctions in a comparative format, highlighting structural and functional differences between reptilian and mammalian vagal systems. By organizing the features side-by-side, the table makes clear that mammalian vagal regulation is not a simple modification of reptilian patterns but rather an evolutionary innovation enabling rapid, socially tuned autonomic flexibility—a capability most clearly expressed in RSA, the focus of the next section.

**Table 2:** Evolutionary differences in vagal function and structure: reptiles vs. mammals.

**Feature**	**Reptiles**	**Mammals**
NAmb function	Somatomotor only	Somatomotor and visceromotor
Cardioinhibitory origin	DMNX (unmyelinated)	NAmb (myelinated) + DMNX (unmyelinated)
Respiratory–heart rate coupling	Inconsistent; lacks central gating	Robust RSA via NAmb
RSA (per PVT)	Absent	Present
Social modulation of vagal tone	Minimal	Extensive; contingent on behavior and social context
Functional integration	Limited	Integrated with vocalization and facial expressivity
Transcriptomic markers	Undocumented	Present (e.g., Mbp, Snap25, and Myrf)

### 13.4 RSA as a signature of mammalian regulation

RSA exemplifies this adaptation. Far from being a passive byproduct of respiration, RSA is a specialized output of a mammalian cardiopulmonary oscillator, integrating respiratory rhythm (pre-Bötzinger complex), cardioinhibitory motor control (NAmb), and diaphragmatic drive (phrenic nerve). While some reptiles exhibit respiratory-linked heart rate changes, these lack central coordination via the NAmb. Thus, RSA is a mammalian innovation—absent in non-mammalian species and dependent on myelinated vagal pathways.

### 13.5 The evolutionary logic of polyvagal theory

Although the vagus nerve is anatomically homologous across vertebrates, it is a categorical error to assume that this structural continuity implies functional equivalence. Gross anatomical homology—such as the presence of the vagus nerve or NAmb—does not capture the profound evolutionary repurposing that defines mammalian autonomic regulation.

Polyvagal theory emphasizes that mammals evolved a nervous system with novel integrative capacities, dynamically coupling physiological state with social behavior. This reorganization—evident anatomically, functionally, and at the level of gene expression—underpins a uniquely mammalian capacity for co-regulation, safety signaling, and flexible social engagement. Critics relying solely on anatomical comparisons overlook these innovations and their molecular correlates.

Emerging transcriptomic analyses reveal that mammalian NAmb neurons express a distinct molecular signature absent in non-mammalian vertebrates. These patterns—discussed in the following section—highlight the specialized role of the VVC in supporting rapid, adaptive autonomic regulation in service of sociality.

### 13.6 The vagal brake and adaptive flexibility

The vagal brake—central to PVT—refers to the VVC's capacity to rapidly inhibit cardiac output via myelinated efferents. When engaged, it promotes calm states conducive to attentiveness, spontaneous social engagement, and co-regulation. Upon detection of threat, the brake is withdrawn, disinhibiting sympathetic activation and enabling rapid mobilization. This bidirectional regulation supports swift shifts between safety and defense, ensuring behavioral flexibility critical to mammalian survival.

In contrast, reptiles—lacking a myelinated ventral vagus—do not possess this dynamic braking system. Their cardiac output is regulated primarily by metabolic demand, without real-time modulation by social cues or brainstem gating. This limits them to slower, less flexible autonomic shifts, marking a key phylogenetic divergence in state regulation and sociality (Porges, 2021).

### 13.7 The social engagement system

The mammalian VVC is an integrated brainstem circuit that links myelinated cardioinhibitory output from the NAmb with the branchial motor (special visceral efferent) nuclei regulating the striated muscles of the head and face. Within this network, cranial nerve (CN) VII controls facial expression and the stapedius muscle; CN V controls mastication and the tensor tympani; CN IX/X (via the NAmb) control laryngeal and pharyngeal muscles for vocal prosody; and CN XI contributes to head orientation.

Although these branchial motor pathways are not NAmb efferents themselves, they are functionally coordinated with NAmb-mediated cardiac regulation, forming a neurophysiological substrate for the social engagement system. This integration enables rapid, context-sensitive shifts between mobilization and calm during socially salient interactions.

In addition to these motor functions, the visceromotor fibers from the NAmb to the heart are uniquely myelinated—a mammalian innovation that allows fast, precise heart rate modulation. This refinement permits fluid physiological adjustments during social interaction, such as rapid deceleration of heart rate during affiliative contact or dynamic tuning during vocal communication.

The middle ear muscles, for example, adjust acoustic transfer properties to preferentially amplify human vocal frequencies, improving the neuroceptive detection of safety cues in the human voice. Through such coordinated adjustments, mammals can both broadcast their autonomic state and perceive it in others, supporting co-regulation, trust, and relational stability. The core elements of this integrated system are summarized in Text Box 2, which outlines the neurophysiological foundations of social engagement.

Box 2 **Social-relational neurophysiology.**

- **Ventral vagal complex (VVC)**: a uniquely mammalian brainstem network composed of interconnected nuclei derived from the branchial arches, including those regulating cranial nerves V, VII, IX, X, and XI. The VVC coordinates cardiac regulation, facial expression, vocalization, and head movement to support adaptive shifts in autonomic state.
- **Social engagement system (SES)**: the behavioral expression of VVC output—manifested in prosody, eye contact, facial affect, and head orientation—that signals safety and facilitates reciprocal connection.
- **Co-regulation**: reciprocal autonomic stabilization via social cues—supporting physiological synchrony and emotional resilience in safe relationships.
- **Vagal brake**: the **cardioinhibitory output** of the VVC—mediated by myelinated vagal fibers from the nucleus ambiguus to the sinoatrial node—enabling rapid heart rate deceleration to support calm states and social engagement.

Collectively, these pathways form the structural foundation for a system that links physiological regulation with social communication—a signature feature of the mammalian autonomic nervous system. The SES is composed of a coordinated set of cranial nerve pathways connecting visceromotor regulation of the heart with branchial and somatic motor control of the face, head, and neck, as well as middle-ear tuning and laryngeal–pharyngeal function.

These relationships are most clearly summarized in Table 3, which lists the primary cranial nerve components of the SES, their functional roles, and their clinical significance.

**Table 3:** Cranial nerve contributions to the social engagement system.

**Component**	**CN**	**Function**	**Clinical significance**
Facial muscles	VII	Expression	Emotion signaling
Middle ear	V, VII	Acoustic tuning	Optimizing speech processing, dampening background sounds
Larynx/pharynx	IX, X	Vocalization	Intonation, signaling safety
Mastication	V	Ingestion	Articulation
Neck/head	XI	Orientation	Social referencing
Heart	X (NAmb)	Heart rate regulation	HRV, resilience

### 13.8 Functional integration and clinical implications

The social engagement system enables co-regulation through coordinated control of expression, vocal tone, auditory filtering, cardiac modulation, and ingestion. This fosters neuroception of safety, downregulating defense circuits and enhancing social accessibility.

Transcriptomic studies confirm elevated expression of genes such as Mbp, Myrf, and Snap25 in NAmb neurons, highlighting the VVC's specialization for rapid, efficient signaling. Disruption of VVC function is associated with difficulties in emotional expression, acoustic sensitivity, social reciprocity, and autonomic regulation—features often observed in conditions such as autism, PTSD, and social anxiety.

Clinical interventions such as the Safe and Sound Protocol (SSP) (Porges and Onderko, 2025) target this system using acoustic input to engage middle ear muscles, enhance vagal tone, and improve social responsiveness. PVT thus frames the social engagement system as a dynamic interface between physiology and behavior—an interface increasingly understood not only anatomically and functionally, but also through the molecular architecture of the VVC.

## 14 Molecular specialization of the mammalian ventral vagal complex (VVC)

The VVC, centered in the NAmb, is both anatomically distinct and molecularly specialized. Comparative transcriptomic analyses reveal that the NAmb expresses a unique constellation of genes absent from homologous brainstem structures in non-mammalian vertebrates. These molecular innovations form the neurobiological substrate for the VVC's hallmark function: rapid, context-sensitive autonomic regulation in support of social engagement.

### 14.1 Transcriptomic differentiation of the NAmb

Several genes are selectively enriched in the mammalian NAmb, contributing to its specialized role in coordinating autonomic and behavioral responses:

- *Mbp* and *Myrf* : Support myelination and high-fidelity conduction along vagal efferents.
- *Snap25*: Ensures precise parasympathetic neurotransmission through regulated vesicle release.
- While oxytocin and vasopressin modulate NAmb activity via descending hypothalamic projections, current transcriptomic evidence does not support direct enrichment of *Oxt* or *Avp* genes in NAmb neurons.

This configuration positions the NAmb as a critical integrator of autonomic regulation and behavioral expression, particularly in mammals where the social engagement system is dependent on precise visceral and somatomotor coordination.

### 14.2 Neuropeptidergic modulation of autonomic state

In mammals, this region-specific expression of neuropeptide receptors uniquely expands the functional repertoire of vagal brainstem nuclei—a specialization not observed in non-mammalian vertebrates.

- **Oxytocin receptors (Oxtr):** Expressed in vagal nuclei such as the dorsal motor nucleus of the vagus (DMNX) and nucleus tractus solitarius (NTS). Their activation supports physiological states marked by social calmness and affiliative engagement requiring immobilization without fear (Carter et al., 2020; Grinevich et al., 2016; Yoshida et al., 2009).
- **Vasopressin receptors (Avpr1a):** More prominently expressed in the dorsal vagal complex and NTS, Avpr1a activation promotes autonomic mobilization in response to perceived threat (Donaldson and Young, 2008).

This receptor distribution supports the state-dependent model of polyvagal theory: Oxytocin facilitates parasympathetic dominance during safety and social bonding, while vasopressin shifts the system toward sympathetic activation under threat.

### 14.3 The NTS as a gateway to body awareness: integrating neuroception and interoception

The NTS serves as a bidirectional integrator of visceral sensory input and a critical modulator of vagal output. Its transcriptomic architecture reflects dense neuropeptide signaling, synaptic plasticity, and rich connectivity with limbic and hypothalamic structures—features that position it as a central node in maintaining homeostasis.

In the framework of polyvagal theory, four key constructs anchor the NTS's role in adaptive regulation:

- **Neuroception** refers to the subcortical detection of cues of safety, danger, or life threat, triggering autonomic shifts without conscious awareness. The NTS participates in this rapid appraisal by relaying sensory input from baroreceptors, chemoreceptors, and visceral afferents to brainstem and forebrain regions that coordinate state changes (Porges, 2003, 2004).
- **Interoception** describes the perception and subjective awareness of internal bodily rhythms, such as respiration, heart rate, and gut motility. Through ascending visceral afferents, the NTS links these signals to cortical structures (e.g., insula and anterior cingulate), creating an embodied representation of physiological state (Porges, 1993).
- **Stress**, in this context, reflects a disruption of homeostatic rhythms caused by instability in the neural feedback circuits that support autonomic regulation—instability that the NTS is uniquely positioned to detect and influence (Porges, 2022).
- **Neurobiological safety** emerges when autonomic feedback loops are stable and integrated, enabling both self-regulation and co-regulation with others (Porges, 2022).

Through its afferent and efferent projections to the NAmb and DMNX, the NTS dynamically modulates autonomic output to support these functions. Neuroception initiates adaptive reactivity, while interoception fosters awareness-based regulation—together allowing the organism to both detect and appraise internal state shifts. By integrating these processes, the NTS acts as a gateway between sensory detection, homeostatic stability, and the flexible state regulation essential for health and social engagement. These principles are distilled in Text Box 3, which summarizes the anchors of homeostasis and the disruptors that compromise it.

Box 3 **Anchors and disruptors of homeostasis.**

- **Neuroception**: subcortical detection of safety, danger, or life threat triggers autonomic shifts without conscious awareness.
- **Interoception**: perception of internal bodily rhythms supports embodied regulation and self-awareness.
- **Stress**: disruption of homeostatic rhythms caused by instability in neural feedback circuits that support autonomic regulation.
- **Neurobiological safety**: emergent from system-wide autonomic stability, enabling regulation, co-regulation, and health restoration.

### 14.4 Functional and clinical implications

The evolutionary repurposing of vagal circuits for social engagement is evident in the VVC's molecular architecture. Disruptions in myelination, synaptic signaling, or neuropeptide expression have been linked to clinical conditions characterized by impaired social functioning and autonomic dysregulation—including PTSD, autism spectrum disorder, and functional somatic syndromes.

Therapeutic approaches targeting the VVC aim to restore autonomic flexibility and enhance co-regulatory capacity. These include the following:

- Vagus nerve stimulation (VNS): Invasive and non-invasive methods to modulate vagal tone.
- Acoustic therapies (e.g., SSP): Stimulate middle ear and vocal tract muscles to facilitate prosodic signaling and social receptivity.
- Respiratory interventions: Use breath pacing and extended exhalation to recruit ventral vagal activity.
- Polyvagal-informed therapies: Leverage relational safety cues and somatic awareness to reactivate ventral vagal regulation.

By engaging the bidirectional properties of the VVC, these interventions promote resilience, coregulation, and recovery through biologically embedded pathways of connection.

## 15 Therapeutic applications of acoustic neuromodulation

Through the development of the Safe and Sound Protocol^®^ (SSP) and the Rest and Restore Protocol™ (RRP), PVT principles have been translated into accessible, sound-based interventions designed to shift autonomic state toward safety, connection, and homeostatic regulation. Grounded in the neurophysiological architecture of PVT, these protocols leverage auditory cues to modulate the social engagement system, targeting neural pathways involved in auditory perception, vocalization, and visceromotor tone. Although differing in their technological implementation, both the SSP and RRP share a common goal: to engage and condition neural circuits that support autonomic flexibility, social behavior, and physiological resilience. Both acoustic interventions are distributed by Unyte Health. Additional information, including supporting research, is available on the Unyte Health website.

### 15.1 Safe and sound protocol (SSP)

The SSP is tightly linked to the social engagement system, particularly its capacity for reflexive, state-dependent modulation of striated muscles involved in vocalization, listening, and head orientation. The SSP employs filtered, prosodically enriched vocal stimuli tuned to the frequencies of human communication. This acoustic stimulation engages cranial nerves V, VII, IX, X, and XI, targeting the neural regulation of striated muscles of the face, head, and neck.

Specifically, the SSP recruits middle-ear muscles—including the tensor tympani (CN V) and stapedius (CN VII)—as well as laryngeal and pharyngeal pathways innervated by cranial nerves IX and X. Importantly, cranial nerve XI supports the dynamic control of head position via the sternocleidomastoid and trapezius muscles, enabling orientation toward vocal cues and optimizing auditory reception. These coordinated components collectively support the modulation of both expressive and receptive aspects of social communication.

The SSP was designed to function as a neural exercise that trains the nervous system through repeated exposure to prosodic auditory cues, reinforcing its ability to detect and respond to signals of safety. This structured engagement acts as a physiological workout, strengthening the neural pathways that support social engagement and autonomic regulation. Peer-reviewed research has shown its effectiveness across a range of clinical populations. In individuals on the autism spectrum, studies have reported improvements in auditory filtering, social engagement, digestive regulation, and vagal function (Porges et al., 2013, 2014).

Recent findings from Kawai et al. (2023), Grooten-Bresser et al. (2024), and Heilman et al. (2023) demonstrate significant improvements in social awareness, anxiety reduction, sleep quality, and eating behaviors, along with a notable decrease in symptoms associated with functional neurological disorders, as further supported by Rajabalee et al. (2022).

### 15.2 Rest and restore protocol (RRP)

While the SSP provides a structured auditory intervention rooted in the human voice and middle-ear activation, the RRP expands therapeutic scope through Sonic Augmentation Technology. Co-developed with Anthony Gorry, Sonic Augmentation Technology embeds neuromodulatory cues within musical compositions to influence core autonomic processes—particularly those synchronized with endogenous physiological rhythms.

Produced and distributed by Sonocea^®^, this technology is designed to signal the nervous system to enter a state of calm immobilization without fear—a foundational neurophysiological state associated with restoration, growth, and healing. This state recruits dorsal vagal pathways that support homeostatic functions. Unlike interventions that rely on active cognitive engagement, the RRP—and other applications of Sonic Augmentation Technology—operates through subconscious pathways, promoting homeostatic regulation passively and efficiently.

The RRP is currently deployed as an adjunctive clinical tool for therapists supporting clients with autonomic dysregulation and sensory hypersensitivities. At the same time, consumer-facing applications are in development, with initial products tailored for neurodivergent individuals—a population often characterized by unstable autonomic regulation. These innovations aim to extend the reach of Polyvagal-informed care, embedding therapeutic modulation into daily life through immersive soundscapes.

Together, the SSP and RRP illustrate how acoustic inputs can be strategically harnessed to target brainstem mechanisms, enhancing vagal tone, auditory receptivity, emotional expressivity, and physiological resilience. By leveraging the biological pathways of the social engagement system and expanding the range of dorsal vagal pathways to support immobilization without fear, these protocols exemplify the translational potential of PVT to reshape nervous system function in ways that are both clinically impactful and ecologically embedded.

## 16 Neurophysiological hierarchies and phylogenetic revisions

The ANS is hierarchically organized in a manner that reflects its evolutionary history. When I introduced PVT, my goal was to illuminate how mammals rely on a phylogenetically derived neural system—the VVC—to support social behavior, emotional nuance, and reciprocal autonomic regulation. Rooted in the NAmb, the VVC gives rise to myelinated vagal efferents that regulate the heart and the striated muscles of the face and head. This mammalian-specific innovation enables rapid, state-dependent modulation of autonomic function, allowing mammals to express and respond to social cues through integrated control of visceromotor, vocal, and facial systems.

The VVC is a mammalian-specific neuroanatomical system absent in non-mammalian vertebrates, including reptiles and birds. These other vertebrate classes lack the myelinated vagal pathways originating in the nucleus ambiguus and the integrated facial motor control necessary to support the uniquely mammalian suite of social engagement behaviors. The integrated VVC—and the myelinated vagus originating from the NAmb—represents synapomorphies of Mammalia: evolutionary innovations that arose after divergence from the common amniote ancestor.

Consequently, the adaptive behavioral repertoire linked to the VVC should not be generalized beyond mammals.

Canonical mammalian synapomorphies include hair or fur, mammary glands, and the diaphragm. To these can be added the suck–swallow–breathe–vocalize circuit, which depends on the VVC to support nursing and social bonding. The emergence of the VVC provided a neurobiological substrate for mammal-specific behaviors such as nurturing, vocal communication, affiliative bonding, and biobehavioral coregulation. By linking visceral state regulation to social signaling, the VVC illustrates how neural structures evolve to sustain complex social ecologies.

When I introduced PVT, my goal was to illuminate how mammals depend on recently evolved neural structures—particularly the VVC—to support social behavior, emotional nuance, and reciprocal regulation. These circuits exert a calming influence on older defense systems but are also the most vulnerable to disruption in the face of threat. This phylogenetic perspective provides a framework for understanding how shifts in neural state can underlie both adaptive and maladaptive behaviors. Rather than viewing clinical symptoms as failures, we can recognize them as expressions of an underlying autonomic strategy—a system reverting to earlier survival mechanisms when safety is compromised.

In conditions of safety, the VVC supports behaviors such as prosodic vocalization, facial expressivity, and listening. If safety cues become ambiguous, the sympathetic nervous system mobilizes the body for fight-or-flight. Under severe or prolonged threat, the system may regress further: The unmyelinated dorsal vagal complex takes over, initiating shutdown, immobilization, or contributing to dissociation. This phylogenetic regression explains why individuals lose access to social skills during periods of stress. Flattened vocal prosody, reduced auditory discrimination, and diminished expressive range are not behavioral flaws—they are neurophysiological adaptations to environments that the nervous system has reflexively determined to lack cues of safety.

Understanding this evolutionary model has fundamentally reshaped my perspective. Rather than focusing exclusively on behavior, emotion, or cognition, I proposed that therapeutic engagement should begin with the regulation of physiological state as its foundational principle. Biomarkers—such as RSA, which reflects myelinated vagal efferent output from NAmb—alongside biobehavioral indicators such as facial expressivity, auditory hypersensitivities, and vocal prosody, offer objective insights into autonomic state. These markers not only reveal underlying physiological processes but can actively guide clinical decision-making by tracking state transitions and informing the timing and type of intervention. This state-informed approach enables more personalized care and optimizes therapeutic outcomes.

The hierarchical organization of the ANS reveals a fundamental insight: Co-regulation is not optional—it is a biological imperative. Recovery from trauma, the maturation of neurodevelopmental capacities, and the healing of emotional wounds all depend on the ability to shift from defensive physiological states into those that support safety, connection, and restoration. Grounded in this framework, my work has focused on developing interventions that facilitate access to the VVC—the neural platform for relational safety, social engagement, and healing.

In both my research and the application of the tools I have developed, my objective has been to provide a roadmap—one that begins with the evolutionary logic of our nervous system, incorporates real-time measures of autonomic function and observations of voice and face, and culminates in relational safety and human connection. As we continue to refine and expand interventions such as SSP and RRP, we move closer to the goal of helping individuals feel safe enough to connect, express, and thrive.

## 17 Responding to critiques and clarifying misrepresentations

Scientific progress depends on rigorous, good-faith engagement with both theory and evidence. Several published critiques of PVT—notably those by Grossman, Taylor, and collaborators—have, at times, mischaracterized the theory's core principles. These critiques often employ strawman arguments, selective citation practices, or overlook empirical developments that address their stated concerns (see Porges, 2007b, 2023).

### 17.1 Major criticisms of polyvagal theory and empirical refutations

PVT has sparked both broad interest and critical debate. Many objections arise not from contradictory data but from misunderstandings of its evolutionary rationale, neuroanatomical specificity, or integrative scope. Table 4 presents these frequently cited criticisms alongside concise, evidence-based responses. This side-by-side format provides context for the more detailed explanations that follow, clarifying how PVT's conceptual and empirical framework addresses—and often preempts—such concerns.

**Table 4:** Common critiques of polyvagal theory and empirical rebuttals.

**Criticism**	**Response**
PVT inaccurately claims that the nucleus ambiguus (NAmb) is unique to mammals.	PVT posits that while the NAmb exists across vertebrates, the mammalian form is distinct in its integration of myelinated vagal efferents and coordination with cranial motor pathways essential for social engagement.
RSA is confounded by respiration and cannot reflect vagal activity.	PVT interprets RSA as a functional output of myelinated vagal pathways, modulated by respiratory rhythms. This modulation reflects an adaptive coupling of cardiac and respiratory systems, not a confound.
Cardiorespiratory coupling is not exclusive to mammals.	PVT acknowledges that non-mammalian vertebrates exhibit cardiorespiratory coupling; however, it differentiates mammalian RSA based on its mediation by myelinated NAmb efferents and coordination through central brainstem oscillators.
PVT overemphasizes the parasympathetic system and neglects sympathetic contributions.	PVT articulates a hierarchical autonomic model in which sympathetic activity is integral to transitions between physiological states, situated between ventral vagal (VVC) and dorsal vagal regulation.
The theory lacks direct empirical support.	Empirical data suggest consistent support for PVT principles across developmental, behavioral, and clinical contexts, including RSA modulation, social engagement behaviors, and responses to threat.
Concepts like “neuroception” and “vagal brake” lack mechanistic specificity.	PVT introduces these constructs as testable hypotheses grounded in neurophysiological organization and supported by behavioral and clinical findings; ongoing research continues to refine their operationalization.
Comparisons between mammalian RSA and reptilian cardiorespiratory coupling suggest functional equivalence.	PVT differentiates these systems based on neuroanatomy and conduction properties; mammalian RSA is proposed to reflect a faster, more flexible mechanism for state regulation.

A recurring issue is the misinterpretation of core concepts. For example, some critiques claim—incorrectly—that PVT asserts the NAmb is exclusive to mammals. In fact, the theory specifies that it is not the mere presence of the NAmb but rather the emergence of the VVC—characterized by myelinated cardioinhibitory efferents originating in the NAmb and integrated with special visceral efferent pathways regulating the striated muscles of the face and head—that distinguishes mammals. This coordinated neuroanatomical architecture, first outlined in Porges (1998), provides the functional substrate for the social engagement system, enabling the co-regulation of autonomic state through facial expressiveness, vocal prosody, and reciprocal social behaviors. Non-mammalian vertebrates lack this system-wide integration, including the necessary myelinated vagal output and cranial nerve coordination, and are thus incapable of the nuanced co-regulatory behaviors supported by the mammalian VVC.

Within this framework, the VVC constitutes a mammalian synapomorphy—a derived neuroanatomical feature that evolved following divergence from a common amniote ancestor. Just as hair and the diaphragm define mammalian clades, so too does the suck–swallow–breathe–vocalize circuit, which depends on the VVC to support nursing, affective vocalization, and affiliative behaviors. The diaphragm, another mammalian synapomorphy, facilitated the precise respiratory control needed for these functions. By linking visceral regulation with social signaling, the VVC exemplifies how neural innovations supported the emergence of complex mammalian sociality.

Some critiques also conflate non-mammalian cardiorespiratory coupling with mammalian RSA, overlooking key differences in neuroarchitecture and evolutionary lineage. While RSA is influenced by respiratory rhythms, this is not a methodological artifact but a defining feature of a functional system. It reflects the dynamic coupling between respiration and vagal efference that facilitates adaptive physiological regulation.

This coupling is mediated by a common cardiopulmonary oscillator (Richter and Spyer, 1990)—a brainstem network that coordinates rhythmic respiratory and cardiac outputs. This oscillator provides a mechanistic substrate for understanding how NAmb-mediated vagal tone interacts with respiratory cycles to support behavioral flexibility and state regulation. In mammals, this architecture enables coordination of breathing with ingestion, vocalization, and social signaling—functions essential for survival and affiliation.

Although respiration–heart rate oscillations are observed in non-mammalian vertebrates (e.g., reptiles and fish), these patterns are analogous rather than homologous to mammalian RSA. They arise from distinct neural substrates lacking the myelinated vagal fibers and cranial nerve integration found in mammals. Therefore, such patterns do not inform the mechanisms or adaptive significance of RSA within the mammalian lineage.

Criticisms asserting that PVT neglects sympathetic contributions also misrepresent the theory's framework. PVT explicitly incorporates sympathetic function within a hierarchically organized model of autonomic regulation, which includes the VVC, sympathetic nervous system, and dorsal vagal complex. This model explains shifts across calm, mobilized, and immobilized states, accounting for sympathetic modulation of arousal, affect, and defensive behavior.

An additional concern is the selective use of PVT-derived constructs—such as neuroception, the vagal brake, and state-dependent regulation—without attribution. Some critiques endorse empirical observations aligned with PVT (e.g., RSA suppression during challenge) while omitting the theoretical framework that confers interpretive coherence. This approach overlooks key principles such as the Jacksonian hierarchy of dissolution and PVT's neuroanatomically informed model of state transitions. By isolating findings from their theoretical foundation, such critiques risk reducing PVT to a disjointed list of observations rather than acknowledging it as a coherent, evolutionarily grounded model of neurophysiological regulation. This not only distorts the theory but also impedes constructive scientific discourse.

### 17.2 Addressing recurrent mischaracterizations of polyvagal theory

Critiques of PVT—notably Grossman and Taylor (2007)—have often been cited as though they represent comprehensive refutations. However, several of their claims were addressed in a contemporaneous response (Porges, 2007b), which clarified areas where aspects of the theory were either misinterpreted or incompletely represented. Central to PVT is the identification of RSA as the functional output of myelinated vagal efferents originating in the NAmb—a distinction foundational to its neurophysiological model of autonomic regulation.

Importantly, PVT does not assert that cardiorespiratory coupling is exclusive to mammals. As stated in the 2007 response:

“…the specific restriction of cardiorespiratory coupling to mammals was not stated in the Polyvagal Theory. Moreover, as discussed in the commentary, from the Polyvagal perspective, RSA is a uniquely mammalian cardiorespiratory interaction because it is dependent on the outflow of the myelinated vagus originating in the nucleus ambiguus. This does not preclude cardiorespiratory interactions involving the unmyelinated vagus originating in the dorsal motor nucleus of the vagus in other vertebrates.” (Porges, 2007b, p. 302)

This distinction is essential. While non-mammalian vertebrates may exhibit respiratory–cardiac interactions via unmyelinated vagal pathways from the DMNX, PVT characterizes mammalian RSA as a phylogenetically derived mechanism that enables rapid, context-dependent modulation of physiological state.

Notably, some elements within the Grossman and Taylor critique appear to align with PVT's foundational premises. For example, their description of RSA as “the final vagal effect on cardiac activity” is consistent with PVT's view of RSA as an index of vagal efference. Similarly, their characterization of RSA as an “energy reserve” echoes the early formulation of the “vagal brake.” These points of conceptual convergence suggest potential areas for productive dialogue. However, when such overlaps are framed as contradictions without acknowledgment of alignment, the opportunity for constructive scientific discourse is diminished.

A related issue arises in Taylor et al. (2022), where comparisons are drawn between DMNX-mediated cardiorespiratory coupling in reptiles and mammalian RSA. From a Polyvagal perspective, this juxtaposition reflects a conflation of functional analogy with neuroanatomical homology. Although both systems involve coordination between respiratory and cardiac rhythms, only mammals possess the myelinated vagal efferents originating in the NAmb and the central oscillator mechanisms (e.g., the pre-Bötzinger complex; Richter and Spyer, 1990) that support rapid, state-dependent modulation. In contrast, reptilian systems lack these features and therefore cannot generate the context-sensitive flexibility characteristic of mammalian RSA. Clarifying these distinctions is critical for accurately representing the evolutionary framework articulated by PVT.

Finally, critiques should acknowledge and engage with prior clarifications—such as Porges (2007b, 2023)—that directly address the conceptual and empirical issues they raise. Omitting these sources risks perpetuating misinterpretations and obscuring the theory's evidentiary foundation. Comprehensive engagement with the full body of PVT literature is essential for maintaining scientific rigor and fostering accurate, productive scholarly discourse.

### 17.3 Clarifying the evolutionary and anatomical basis of PVT

PVT characterizes the mammalian NAmb as an evolutionary specialization distinct in function from homologous brainstem structures in non-mammalian vertebrates. Although motor nuclei involved in visceromotor control are broadly conserved across species, the mammalian NAmb is marked by the emergence of myelinated vagal efferents. These pathways facilitate rapid and metabolically efficient regulation of visceral organs and striated muscles integral to social communication (Porges, 2007a, 2023). This specialization supports the coordinated control of facial expressions, vocalization, and cardiac output—key components of the mammalian social engagement system.

Recent single-cell transcriptomic atlases of the mouse nervous system (e.g., Zeisel et al., 2018) provide foundational region- and cell-type-specific gene expression frameworks. While early efforts to resolve the molecular identity of the nucleus ambiguus (NAmb) were limited, emerging studies have now delineated its transcriptomic signature. Coverdell et al. (2019) first employed single-cell profiling to disambiguate NAmb neurons from adjacent medullary populations, identifying distinct expression of genes associated with fast-conducting, myelinated efferents. Building on this, Jalil et al. (2023) demonstrated that canonical myelination- and synaptic-vesicle-associated transcripts—such as *Mbp, Myrf*, and *Snap25*—are selectively enriched in NAmb neurons, suggesting a molecular substrate for rapid vagal efferent conduction and precise cardiovagal modulation. *Snap25's* essential role in precision exocytosis has been independently demonstrated in sensory systems, including auditory synapses (Goel et al., 2022). In contrast, neurons in the dorsal motor nucleus of the vagus (DMNX) exhibit a neuromodulatory profile consistent with slower, unmyelinated parasympathetic projections (Hornung et al., 2024). These findings are further supported by integrative circuit-level analyses from Veerakumar et al. (2022), who mapped distinct cardiopulmonary efferent pathways, underscoring the anatomical and functional specialization of the NAmb.

In addition to structural and molecular adaptations, mammals exhibit distinct neuropeptidergic profiles within brainstem autonomic circuits. While the NAmb itself does not produce oxytocin (*Oxt*) or vasopressin (*Avp*), their receptors (*Oxtr* and *Avpr1a*) are prominently expressed in neighboring nuclei, including the dorsal motor nucleus of the vagus (DMNX) and nucleus tractus solitarius (NTS). These circuits participate in modulating autonomic state in response to social and environmental cues, enabling transitions across mobilized, immobilized, and affiliative states (Donaldson and Young, 2008; Grinevich et al., 2016).

The broader distribution and functional expansion of oxytocinergic and vasopressinergic systems in mammals may represent a synapomorphic feature—supporting social bonding, co-regulation, and behavioral resilience (Lee et al., 2009; Insel et al., 2010). Their interaction with brainstem regulatory circuits further distinguishes mammalian autonomic organization from that of earlier vertebrate lineages.

Taken together, the NAmb and its affiliated networks illustrate a coordinated set of evolutionary modifications—encompassing gene expression, neuroanatomy, and neurochemical signaling—that enable the flexible social behaviors emphasized in PVT.

### 17.4 Empirical foundations, testable predictions, and translational metrics

PVT has yielded several testable hypotheses and validated across developmental, clinical, and psychophysiological research:

- Hierarchical autonomic response sequence: a core prediction of PVT—sequential recruitment of autonomic subsystems under threat—is supported by physiological and behavioral data. This sequence begins with ventral vagal engagement (supporting social behavior and calm), shifts to sympathetic mobilization (fight/flight), and culminates, under extreme threat, in dorsal vagal shutdown (immobilization without social engagement) (Porges, 1995, 2007a, 2023).
- RSA recovery as a resilience index: the speed of post-stress recovery of RSA is predictive of emotion regulation, physiological resilience, and flexible behavioral responding. This measure has been validated across clinical and developmental populations (Beauchaine, 2001; Balzarotti et al., 2017).
- Vagal efficiency as a clinical biomarker: vagal efficiency, defined as the dynamic coupling between RSA and heart rate, reflects the effectiveness of cardiac vagal regulation. It has emerged as a translational biomarker in studies of trauma (Dale et al., 2022), anxiety, depression, and neurodevelopmental conditions (e.g., Porges, 2025).

Importantly, the Porges–Bohrer method for RSA quantification has addressed prior methodological concerns by minimizing respiratory confounds and accommodating signal non-stationarity (Lewis et al., 2012). The associated patent (Porges, 1985a,b) has been cited in over 500 peer-reviewed studies.

In clinical applications, vagal efficiency—quantified using the Porges–Bohrer method—has shown predictive value in pediatric pain and nausea trials (Kovacic et al., 2020; Kolacz et al., 2025), underscoring its utility as a physiologically grounded, psychometrically validated index of autonomic flexibility.

### 17.5 Neuropeptidergic modulation as an evolutionary extension

PVT originally proposed that neuropeptides such as oxytocin and vasopressin modulate autonomic state by acting on vagal brainstem nuclei (Porges, 2001). This hypothesis has gained support from recent transcriptomic studies identifying oxytocin and vasopressin receptor expression in key autonomic regions—most notably the nucleus of the NTS and dorsal motor nucleus of the DMNX. These neuropeptidergic systems are thought to contribute to flexible biobehavioral transitions in mammals between states of social engagement and defensive reactivity.

For example:

- Oxytocin signaling within vagal brainstem circuits, particularly in the NTS and DMNX, may support facial expressivity, vocal prosody, and affiliative communication, although direct oxytocin receptor expression in the NAmb remains to be fully documented.
- Vasopressin signaling in the DMNX and NTS has been associated with heightened vigilance, autonomic arousal, and defensive withdrawal in response to threat.

This neuromolecular architecture, which integrates neuropeptide sensitivity with brainstem–autonomic pathways, appears to be absent or markedly less developed in reptiles and birds. These findings reinforce the evolutionary specificity of mammalian autonomic regulation as described in PVT and offer direct counterpoints to critiques claiming the theory lacks a comparative or phylogenetic foundation.

### 17.6 Clinical implications and intervention pathways

Disorders marked by impaired ventral vagal access—such as autism, PTSD, and anxiety—are increasingly associated with dysregulated oxytocin and vasopressin systems (Carter et al., 2020). For instance, diminished oxytocin receptor expression may hinder co-regulation, while elevated vasopressin levels may sustain defensive hypervigilance (Cochran et al., 2013; Meyer-Lindenberg et al., 2011). These findings have catalyzed a new generation of interventions targeting brainstem-autonomic pathways, particularly those informed by the functional architecture of the vagus nerve.

Emerging strategies include the following:

- **The safe and sound protocol**™ **(SSP)**: A targeted acoustic intervention designed to engage the VVC and activate the social engagement system through prosodically modulated vocal input. SSP improves extraction of human speech and social cues and promotes a neuroception of safety (Grooten-Bresser et al., 2024; Heilman et al., 2023; Porges et al., 2013, 2014).
- **Sonic augmentation technology**: this broad auditory modulation strategy is designed to influence both the VVC and the DMNX. By supporting transitions into a calm, immobilized state—immobilization without fear—it facilitates the body's endogenous regulation of homeostatic functions, including digestion, anti-inflammatory responses, immune modulation, and physiological restoration.
- **The rest and restore protocol:** a clinical application of Sonic Augmentation Technology, this intervention delivers rhythmically structured, frequency-modified soundscapes designed to entrain parasympathetic activity and enhance vagal efficiency. As an adjunctive therapy, it facilitates the transition from chronic defensive states to restorative autonomic regulation.
- **Vagal nerve stimulation (VNS)**: applied either invasively or non-invasively, VNS directly modulates vagal afferents to recalibrate autonomic function. It has shown efficacy in enhancing vagal tone, reducing sympathetic dominance, and improving resilience across a range of clinical populations.
- **Polyvagal-informed therapies**: These approaches emphasize the importance of cues of safety, reciprocal co-regulation, and attuned therapeutic presence. By shaping the social environment to support ventral vagal activation, they aim to shift the client's physiological state to one conducive to connection, healing, and learning.

Together, these interventions illustrate a translational shift in clinical strategy—from top-down cognitive models to bottom-up neurophysiological regulation, leveraging the vagus nerve as a portal to restore adaptive functioning and relational capacity.

### 17.7 A dual-level framework: evolution and clinical science

PVT bridges proximate mechanisms and ultimate evolutionary functions. For instance, Leontiadis and Longstreth (2020) proposed that PVT offers a compelling model for conditions such as pediatric abdominal pain, where chronic threat detection maintains defensive states (Kolacz and Porges, 2018). By linking vagal disruption to both immediate physiology and adaptive evolution, PVT provides a holistic account of stress-related disorders.

Their editorial on vagal efficiency contextualizes PVT within an evolutionary medicine paradigm, integrating findings on reduced RSA, diminished vagal tone, and trauma-induced disruptions to autonomic regulation. By advancing a dual-level framework—encompassing phylogenetic neuroanatomy and clinical dysregulation—their editorial enhances the explanatory depth of PVT and effectively rebuts critiques that dismiss it as merely descriptive.

## 18 Polyvagal theory: evolutionary insights and core contributions

PVT offers a transformative, interdisciplinary framework for understanding how the ANS supports social engagement, physiological regulation, and adaptive behavior. Grounded in evolutionary biology, neurophysiology, behavioral science, and clinical research, PVT reframes autonomic state not as a background process but as a dynamic mediator of experience, health, and social connection.

### 18.1 Foundational principles

At the core of PVT are several key principles that define the structure and function of the mammalian ANS:

- **Phylogenetic hierarchy:** the ANS evolved in stages. Mammals possess three functionally distinct subsystems that regulate behavior according to detection of signals of risk and safety:
  ° Ventral vagal complex: Social engagement and calm states.
  ° Sympathetic: Mobilization and fight/flight responses.
  ° Dorsal vagal complex: Immobilization and shutdown under extreme threat.
- **Jacksonian dissolution (hierarchical deactivation):** under threat, newer neural systems deactivate first, leading to reactivation of evolutionarily older defensive strategies. This explains behavioral regression seen in trauma or extreme stress.
- **Ventral vagal cardioinhibitory pathway:** unique to mammals, this myelinated pathway (originating in the NAmb) enables rapid, flexible cardiac regulation—underlying RSA and supporting dynamic social engagement.
- **Neuroception:** a neural process that detects cues of safety, danger, or life threat—both in the environment and within the body—and initiates autonomic state shifts without involving conscious awareness.
- **Social engagement system:** a mammalian circuit that integrates special visceral efferent pathways within cranial nerves V, VII, IX, X, and XI to coordinate facial expression, vocal prosody, posture, head orientation, and middle-ear muscle tone. This enables bidirectional signaling and coregulation.
- **Cardiopulmonary oscillator and brainstem coordination:** RSA and weighted coherence are expressions of a mammalian-specific oscillator that synchronizes cardiac and respiratory rhythms—a key innovation in flexible physiological regulation.

### 18.2 A unified empirical framework

Polyvagal theory synthesizes convergent findings across empirical domains:

- **Comparative anatomy:** evolution of dual vagal motor pathways and cranial nerve integration.
- **Neurophysiology:** emergence of RSA and the myelinated ventral vagal brake.
- **Behavioral science:** observation of state-dependent modulation of social signals.
- **Transcriptomics:** molecular specialization of mammalian vagal nuclei (e.g., myelin-related gene expression).
- **Clinical science:** translation into therapy, education, trauma response, and public health.

Together, these fields support a biologically anchored understanding of how mammals detect safety and regulate engagement.

### 18.3 Metrics and innovations

PVT has introduced novel physiological measures and conceptual tools:

- **RSA (Porges–Bohrer method):** a robust index of ventral vagal tone.
- **Vagal efficiency:** quantifies how effectively RSA modulates heart rate in response to environmental demands.
- **Weighted coherence:** measures synchrony between RSA and respiratory rhythm, reflecting brainstem oscillator integrity.
- **Stress as oscillatory breakdown:** redefines stress as a disruption in physiological rhythms, rather than just increased arousal.

### 18.4 Clinical and translational significance

PVT provides a roadmap from biological insight to applied innovation:

- **Clinical applications:** PVT informs trauma therapies, neurodevelopmental interventions, biofeedback, pediatric and perinatal care, and autonomic monitoring.
- **Diagnostic reframing:** conditions such as PTSD, autism, and anxiety are seen not as fixed pathologies but as adaptive expressions of autonomic state dysregulation.
- **Institutional transformation:** PVT guides the redesign of educational, therapeutic, and healthcare settings around the imperative for safety and co-regulation.

## 19 Conclusion and future applications

The development of PVT represents more than five decades of empirical and conceptual work linking autonomic physiology to behavior, emotion, and health. Originating in early research on HRV as an index of autonomic–behavioral reactivity, this body of work progressively advanced toward a comprehensive evolutionary model of autonomic state regulation. Along the way, it identified respiratory sinus arrhythmia (RSA) as a neural marker of vagal regulation, experimentally mapped the brainstem circuits mediating RSA, and reframed stress as a disruption of homeostatic physiological rhythms. The integration of evolutionary phylogeny and Jacksonian dissolution principles into a hierarchical autonomic framework provided a unifying structure for interpreting adaptive and maladaptive responses to environmental challenges. This progression is reflected in the chronological milestones of Polyvagal Theory, summarized in Table 5.

**Table 5:** Chronological milestones in the evolution of polyvagal theory.

**Year range**	**Core feature**	**Key concept**	**References**
1967–1974	HRV as an index of autonomic–behavioral reactivity	Established HRV as both a predictor of autonomic regulation and a correlate of sustained attention and behavioral performance. Demonstrated HRV as a mediator of newborn autonomic reactivity and temporal conditioning, shifting HRV from “noise” to a meaningful physiological marker.	(Porges and Raskin, 1969; Porges, 1972, 1973, 1974)
1975–1984	RSA as a neural marker of vagal regulation	Documented depressed HRV in developmental disabilities; identified RSA as a neurally mediated index of vagal regulation; developed weighted coherence metrics; mapped brainstem vagal pathways in animal models; created the Porges–Bohrer method for quantifying RSA; and applied spectral analysis to fetal and neonatal HRV.	(Menedez-Bauer et al., 1979; Porges and Humphrey, 1977; Porges et al., 1980, 1981; McCabe et al., 1984; Larson and Porges, 1982; Yongue et al., 1982; Donchin et al., 1984)
1985–1994	Foundations for PVT	Redefined stress as a disruption of physiological homeostasis; linked RSA to clinical prognostics (e.g., predicting surgical outcome and anesthesia effects); introduced the concept of interoception (1993) to describe the neural mechanisms underlying awareness of internal bodily states; explored the effects of cholinergic blockade on autonomic regulation; and patented the Porges–Bohrer methodology for quantifying RSA.	(Porges, 1985a,b, 1986, 1993; Donchin et al., 1985, 1992)
1995–2004	Formalization and refinement of PVT	Published the foundational presentation of PVT (1995), introducing the phylogenetic hierarchy of autonomic state regulation, distinguishing ventral and dorsal vagal pathways, and acknowledging Richter & Spyer's model of the common cardiopulmonary oscillator. Introduced the vagal brake (1996); described the social engagement system (1998); integrated the Jacksonian principle of dissolution (2001) to explain predictable regression to evolutionarily older autonomic circuits under challenge; defined vagal efficiency (1999); coined “neuroception” (2003); and tested hypotheses on infant viability and sleep state regulation.	(Porges, 1995, 1998, 2001, 2003; Porges et al., 1996, 1999; Reed et al., 1999)
2005–2014	Translation into therapeutic frameworks	Updated and expanded PVT in *The Polyvagal Perspective* (2007), broadening its theoretical scope and clinical relevance; developed the Listening Project Protocol (precursor to the Safe and Sound Protocol); articulated the bidirectionality of the autonomic hierarchy; advanced polyvagal-informed clinical practices; demonstrated the sensitivity of the Porges–Bohrer method to cholinergic blockade; and documented the link between vocalizations and RSA.	(Kolacz et al., 2022; Porges, 2007a; Porges et al., 2013, 2014; Lewis et al., 2012; Porges and Lewis, 2010)
2015–2025	Consolidation and expansion	Expanded applications of PVT in clinical and translational domains; co-founded the Polyvagal Institute (2020) to promote the integration of polyvagal principles across disciplines through research, training, and international collaboration; refined the scientific basis for vagal efficiency as a biomarker; patented the application of vagal efficiency; patented and licensed the technology underlying scaled acoustic interventions as adjunctive neuromodulators; published *The Vagal Paradox: A Polyvagal Solution* (2023) as a comprehensive exposition of the neuroscience underlying PVT; and documented associations between vagal efficiency and clinical status in dysautonomia and gastroenterology.	(Porges, 2022, 2023, 2025; Kovacic et al., 2020; Kolacz et al., 2021, 2023)

Subsequent refinements introduced concepts that have since become central to both basic and applied science: the vagal brake as a rapid-response mechanism for regulating cardiac output, the social engagement system as an emergent property of the ventral vagal complex, the construct of neuroception to describe implicit detection of safety and threat, and vagal efficiency as a quantifiable index of autonomic flexibility. These conceptual advances provided the scientific foundation for auditory-based interventions such as the Listening Project Protocol and its clinical successor, the Safe and Sound Protocol, as well as polyvagal-informed therapeutic practices emphasizing co-regulation, prosody, and cues of safety.

This timeline traces the theory's development from foundational physiological research to clinical translation, highlighting key conceptual innovations, empirical findings, and applied interventions that together form the cumulative evidence base for PVT. It illustrates how PVT has matured from a framework for interpreting physiological signals to a translational science with direct clinical applications, emphasizing the continuity between foundational research and contemporary innovations. By mapping this progression, the table shows how decades of interdisciplinary inquiry have shaped a connection-centered science of health—providing both a historical record and a framework to guide future research and applications. Building on this foundation, PVT continues to evolve alongside advances in neuroscience, molecular biology, clinical intervention, and public health. Technological developments—from wearable biometrics and real-time signal analysis to transcriptomic profiling—are enhancing our ability to operationalize core PVT constructs, including neuroception, co-regulation, and vagal flexibility. These tools promise not only expanded research precision but also greater translational utility, paving the way for more personalized and connection-centered approaches to health.

### 19.1 Integrated physiological and molecular metrics

As outlined in the preceding timeline, the identification of mammalian-specific vagal pathways and their role in social engagement provides a structural and evolutionary foundation for future research. New technologies now allow for dynamic tracking of autonomic state in naturalistic environments, expanding the precision with which core PVT constructs can be operationalized. These include time–frequency analysis, non-contact biometrics (e.g., pupillometry and photoplethysmography), and wearable-derived indices of vagal efficiency and RSA.

In parallel, transcriptomic studies are beginning to identify gene expression profiles unique to mammalian vagal nuclei—particularly the NAmb and NTS—that support rapid, context-dependent autonomic regulation. These molecular specializations mirror the phylogenetic advances detailed in the timeline and provide a genetic scaffold for PVT functions such as social engagement, behavioral flexibility, and adaptive state regulation. By linking gene expression patterns to physiological outcomes, these approaches offer a new bridge between evolutionary biology and individualized clinical application.

### 19.2 Clinical translation and personalized intervention

The clinical innovations that emerged in the later phases of the timeline—such as the Listening Project Protocol and Safe and Sound Protocol—demonstrate how PVT can be translated into targeted, measurable interventions. Current polyvagal-informed approaches include vagus nerve stimulation, acoustic modulation (e.g., the Safe and Sound Protocol and Rest and Restore Protocol, licensed and distributed by Integrated Listening System/Unyte Health), breath-based practices, and coregulatory psychotherapies.

Sonic augmentation technologies—such as Sonocea's Rest and Restore Protocol—are designed to influence foundational brainstem circuits through precisely modulated auditory cues, tailored in tempo and frequency to support immobilization without fear. These cues may engage subcortical mechanisms, including those arising from the DMNX, to facilitate calm physiological states conducive to restoration and repair.

In contrast, the Safe and Sound Protocol is hypothesized to target the VVC via its influence on middle ear muscle function, thereby enhancing prosody detection, social listening, and co-regulation. This may occur through modulation of efferent pathways of cranial nerves V (trigeminal) and VII (facial), improving the extraction of human vocal signals from ambient noise. By increasing the signal-to-noise ratio for socially relevant acoustic cues, the Safe and Sound Protocol may promote neurophysiological states that support safety and social engagement.

Together, these interventions illustrate how rhythmically and spectrally modulated acoustic inputs can influence neural circuits central to autonomic state regulation, physiological recovery, and neuroception of safety. Biometrics such as RSA and vagal efficiency provide objective markers to evaluate intervention efficacy and refine protocols for personalized delivery.

### 19.3 Social systems and policy implications

The emphasis on safety and co-regulation that runs through PVT's historical development extends beyond clinical contexts to broader social systems. Institutions such as schools, healthcare settings, and justice systems can be restructured to support biological safety and relational trust, aligning environmental conditions with the neurophysiological needs described in PVT.

As polyvagal-informed technologies and practices scale into public health domains, equitable access and cultural responsiveness will be essential to successful implementation. Examples include trauma-informed educational environments, autonomically attuned clinical care, and justice systems designed to reduce physiological threat. Embedding the principles summarized in the timeline into policy and practice may help create systemic conditions that foster resilience, connection, and community wellbeing.

### 19.4 Looking ahead

The trajectory mapped in the preceding timeline demonstrates that polyvagal theory (PVT) evolves most productively when grounded in cumulative evidence and interdisciplinary integration. Future advances will likely emerge from collaborations bridging molecular biology, neurophysiology, behavioral science, and clinical innovation.

Physiological metrics will increasingly leverage real-time biometrics, including wearable sensors, pupillometry, and emerging transcriptomic and neuroimaging approaches to vagal structures—the NAmb, DMNX, and NTS. These developments will enable individualized, non-invasive assessments of autonomic flexibility and resilience, while facilitating integration of biometric indices such as vagal efficiency and RSA with vagal-associated gene expression.

Intervention science will focus on refining and personalizing non-invasive vagal nerve stimulation, coregulatory therapies, and sound- or breath-based protocols, optimizing responsiveness for conditions including trauma, anxiety, neurodevelopmental disorders, and chronic disease. Advances in stress and resilience modeling—through biomarkers such as RSA, vagal efficiency, and weighted coherence—will combine with predictive algorithms to guide precision diagnostics and tailored interventions in behavioral health.

Applying polyvagal principles across education, healthcare, and justice systems could enhance safety, regulation, and support in high-impact environments. Cross-cultural and comparative research, including studies on caregiving, cultural context, ecological variation, and interspecies comparisons, will expand understanding of the ventral vagal complex and sociality, informing culturally attuned interventions.

Ethics and equitable access will remain central to the responsible deployment of polyvagal-informed technologies. Interdisciplinary collaboration—spanning neuroscience, engineering, architecture, education, clinical practice, policy, and other applied disciplines—will be essential for translating PVT into scalable, culturally responsive solutions. By aligning scientific innovation with thoughtful communication and application, the next decade offers the opportunity for PVT to fulfill its promise as both a rigorous scientific framework and a practical guide for connection-centered health.

## 20 Epilog: reflections on science, popularization, and the role of theory

PVT emerged from decades of laboratory-based research conducted within the traditional framework of scientific inquiry—generating hypotheses, testing models, publishing peer-reviewed findings, and presenting at academic conferences. As the preceding timeline illustrates, its trajectory has been cumulative, building step-by-step from foundational physiology to translational applications.

Yet PVT's impact has extended well beyond academic discourse. Clinicians, educators, and wellness practitioners have adopted its concepts, adapting them to diverse contexts. This diffusion underscores both the resonance and the reach of the theory—but also brings challenges. As ideas move into new domains, they are often simplified, reframed, or abstracted in ways that can detach them from their empirical foundations.

This raises an important question for modern science: What is the scientist's role when a theory lives beyond the boundaries of academia? Historically, the flow of knowledge was largely unidirectional—from laboratory to application—with empirical verification as the primary gatekeeper. Today, the relationship is more dynamic. Clinical observations, experiential reports, and intuitive insights increasingly flow back into the theory's public image, sometimes more visibly than published data. Social media amplifies this process, allowing complex ideas to be reshaped, sometimes in ways that misrepresent their original scope.

In this environment, scientific responsibility extends beyond discovery to stewardship—maintaining the precision, integrity, and evidentiary grounding of a theory as it travels across disciplines and into public consciousness. Recent clarifying publications and theoretical updates are not acts of defensiveness but of accountability. They reaffirm the need for clarity as PVT is applied in psychotherapy, neurotechnology, trauma recovery, public health, and beyond.

Mischaracterizations—such as those found in critiques by Grossman and Taylor—illustrate the risks of selective citation and omission of prior clarifications (e.g., Porges, 2007b, 2023). These critiques have often focused on rejecting foundational premises without engaging the full evidentiary record or proposing alternative, testable models. The persistence of such approaches has allowed strawman representations of PVT to proliferate, both in academic literature and in online discourse, obscuring the empirical and theoretical advances the theory has achieved.

Scientific progress depends on structured comparison, where competing explanations are tested against evidence and refined through falsifiable predictions. In this sense, PVT remains a living framework—one that welcomes refinement, invites collaboration, and seeks coherence across biological, psychological, and clinical sciences.

The broader lesson extends beyond PVT. In today's interconnected landscape, theories do not remain static or siloed—they evolve, hybridize, and acquire new meanings as they move across communities. For them to remain scientifically credible and socially valuable, they must be nurtured with both innovation and fidelity to their empirical roots.

In closing, PVT's reach reflects both promise and responsibility. As its insights continue to inform interventions, technologies, and narratives of human experience, its clarity and integrity must be preserved. The trajectory mapped in this study—from foundational discoveries to contemporary clinical tools—demonstrates what is possible when science is practiced with both rigor and openness. In the spirit of Platt's (1964) call for strong inference, the continued vitality of PVT will depend on sustained commitment to hypothesis-driven inquiry, interdisciplinary collaboration, and the disciplined refinement of ideas.

## Glossary

**RSA**, *respiratory sinus arrhythmia*—A naturally occurring respiratory rhythm in the spontaneous fluctuations of beat-to-beat heart rate mediated by ventral vagal tone. The amplitude of RSA is a valid, non-invasive index of ventral vagal activity.

**NAmb**, *nucleus ambiguus*—A brainstem nucleus within the ventral vagal complex that provides myelinated cardioinhibitory vagal output to the heart and motor control to laryngeal and pharyngeal muscles involved in vocalization, swallowing, and other components of social communication, via special visceral efferent pathways within cranial nerves IX and X.

**DMNX**, *dorsal motor nucleus of the vagus*—A brainstem nucleus providing primarily unmyelinated vagal output to thoracic and abdominal organs, involved in metabolic regulation and defensive immobilization.

**VVC**, *ventral vagal complex*—A set of brainstem nuclei, emerging embryologically from the pharyngeal arches, including the nucleus ambiguus. The VVC regulates the heart through cardioinhibitory vagal pathways and coordinates social engagement behaviors, including vocalization, via special visceral efferent pathways within cranial nerves V, VII, IX, X, and XI.

**HRV**, *heart rate variability*—A measure of variation in the time interval between heartbeats, reflecting dynamic autonomic nervous system regulation.

**PVT**, *polyvagal theory*—A neurophysiological model describing how the autonomic nervous system supports adaptive regulation of physiological state, social engagement, and defensive responses, based on evolutionary hierarchies.

**NTS**, *nucleus tractus solitarius*—A brainstem nucleus that integrates visceral sensory input and coordinates autonomic output; central to processes of neuroception and interoception.

**VNS**, *vagus nerve stimulation*—A therapeutic technique, invasive or non-invasive, that stimulates the vagus nerve to influence autonomic state and behavior.
